# Copper-catalyzed domino cyclization of anilines and cyclobutanone oxime: a scalable and versatile route to spirotetrahydroquinoline derivatives

**DOI:** 10.3762/bjoc.21.58

**Published:** 2025-04-09

**Authors:** Qingqing Jiang, Xinyi Lei, Pan Gao, Yu Yuan

**Affiliations:** 1 School of Chemistry and Chemical Engineering, Yangzhou University, Yangzhou 225002, People’s Republic of China; 2 School of Tourism and Cuisine, Yangzhou University, Yangzhou 225002, People’s Republic of China

**Keywords:** copper catalysis, cyclobutane-fused tetrahydroquinolines, domino cyclization reaction, green synthesis

## Abstract

In this study, we report the copper-catalyzed synthesis of tetrahydroquinoline derivatives via a domino reaction of aniline with cyclobutanone oxime. This method demonstrates a selective approach for generating bioactive tetrahydroquinoline scaffolds, which have broad applications in pharmaceutical chemistry. The reaction conditions were optimized for the effective formation of tetrahydroquinoline derivatives with varying substituents, showing high yields under mild conditions. Mechanistic studies suggest a catalytic cycle involving nucleophilic attack by the aniline on the cyclobutanone oxime, followed by cyclization to form the desired product.

## Introduction

**Scheme 1:**
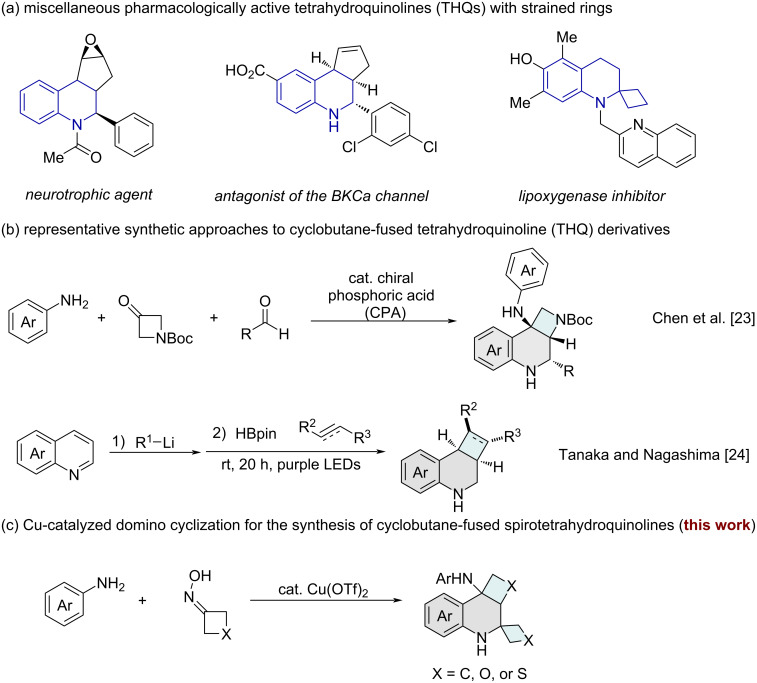
Synthetic strategies for the construction of spirotetrahydroquinoline (STHQ) scaffolds.

Tetrahydroquinolines (THQs) represent a privileged scaffold in medicinal chemistry, exhibiting a broad spectrum of biological activities and serving as pivotal structural elements in drug discovery [1–4]. Notably, tetrahydroquinoline derivatives featuring a strain-inducing ring system are prevalent in numerous bioactive molecules, including those with promising therapeutic potential for neurological disorders, oncology, and various other medical conditions (Scheme 1a) [5–8]. Consequently, the development of efficient synthetic methodologies for constructing fused THQs is of paramount importance for advancing pharmaceutical research. Conventional synthetic strategies for THQs, which involve the formation of highly strained rings, typically employ catalytic cyclization [9–13], reductive aminations [14–17], and photochemical cyclization [18–20]. However, these approaches often necessitate multistep syntheses of starting materials and involve intricate experimental procedures, significantly impeding their practical utility and scalability [9–20].

Cyclobutane-fused tetrahydroquinolines (THQs) have garnered significant attention in drug discovery due to their inherent structural rigidity and enhanced pharmacological profiles [21], which render them highly desirable for therapeutic development. The strained cyclobutane ring, in particular, acts as a versatile and conformationally constrained building block, enabling the construction of complex molecular architectures with potent biological activities [22]. Despite their promise, the synthesis of cyclobutane-fused THQs remains a formidable challenge, primarily due to the inherent ring strain and the difficulties associated with achieving high diastereoselectivity during cyclization [4]. Recently, Chen and co-workers developed a chiral phosphoric acid (CPA)-catalyzed multicomponent reaction of anilines, aldehydes, and azetidinones, enabling the efficient and enantioselective synthesis of tetrahydroquinoline-fused azetidines with three contiguous stereocenters in a single step [23]. Later, Tanaka, Nagashima, and their co-workers established a chemo-, regio-, and diastereoselective dearomative transformation of quinolines into tetrahydroquinoline (THQ)-based 6-6-4-membered ring systems through a combination of nucleophilic addition and borate-mediated [2 + 2] photocycloaddition, offering a catalyst-free approach to construct conformationally constrained 2D/3D frameworks with high functional group compatibility and stereocontrol (Scheme 1b) [24]. In 2023, Zeng et al. reported the first example of a chromium-catalyzed spirocyclization between anilines and cyclobutanones, providing direct access to medicinally relevant cyclobutane-annulated and structurally constrained spirotetrahydroquinoline (STHQ) scaffolds [25]. Given the growing significance of cyclobutane-fused tetrahydroquinolines (THQs) in biochemistry and medicinal chemistry [26–28], we have developed an efficient and convenient method for synthesizing cyclobutane-fused and conformationally constrained spirotetrahydroquinolines (STHQs) from arylamines and cyclobutanone oxime using a copper-catalyzed reaction under ambient air conditions (Scheme 1c).

## Results and Discussion

With these considerations in mind, we explored the feasibility of synthesizing cyclobutane-fused spirotetrahydroquinolines (STHQs) through the reaction of arylamines with cyclobutanone oxime under copper catalysis. After extensive optimization of the reaction parameters, the desired product **3aa** was obtained in 92% yield under the following optimal conditions: the reaction between aniline (**1a**) and cyclobutanone oxime (**2a**) as the model system, hexane as the solvent, and copper(II) trifluoroacetate (Cu(TFA)_2_) as the catalyst (20 mol %) under ambient air at 80 °C for 12 hours; the product **3aa** was isolated by chromatographic purification (Table 1, entry 1). The use of other solvents, including acetonitrile (MeCN), tetrahydrofuran (THF), toluene, acetone and methanol (MeOH), resulted in significantly lower yields of **3aa** (Table 1, entry 2). Replacing the Cu(TFA)_2_ catalyst with other copper sources, such as cuprous chloride (CuCl), cuprous thiocyanate (CuSCN), copper bromide (CuBr_2_), copper trifluoromethanesulfonate (Cu(OTf)_2_), and copper powder resulted in diminished reaction efficiency (Table 1, entry 3). When iron(II) sulfate (FeSO_4_) and iron trifluoromethanesulfonate (Fe(OTf)_2_) were used as the catalyst instead of copper(II) trifluoroacetate (Cu(TFA)_2_), the yields of the product were decreased (Table 1, entries 4 and 5). Using palladium(II) acetate (Pd(OAc)_2_) as the catalyst provided a moderate yield (Table 1, entry 6). Conducting the reaction at room temperature (rt) instead of the optimal elevated temperature resulted in a lower yield (Table 1, entry 7). Increasing the reaction temperature to 100 °C did not improve the yield (Table 1, entry 8).

**Table 1:** Optimization of reaction conditions.^a^

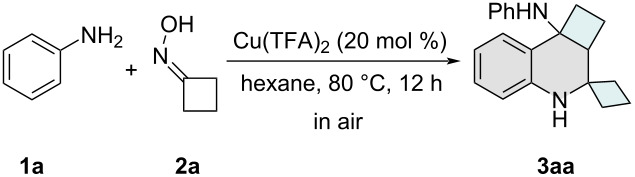		

Entry	Deviation from "standard conditions"	Yield of **2a** (%)^b^

1	none	92
2	MeCN, THF, toluene, acetone, or MeOH instead of hexane	20–70
3	CuCl, CuSCN, CuBr_2_, Cu(OTf)_2_ or Cu powder instead of Cu(TFA)_2_	31–60
4	FeSO_4_ instead of Cu(TFA)_2_	76
5	Fe(OTf)_3_ instead of Cu(TFA)_2_	74
6	Pd(OAc)_2_ instead of Cu(TFA)_2_	67
7	rt	71
8	100 °C	63

^a^Reaction conditions: aniline (**1a**, 0.2 mmol), **2a** (0.4 mmol), and Cu(TFA)_2_ (0.04 mmol) in hexane (2.0 mmol) under air atmosphere, 12 h, 80 °C. ^b^Isolated yields after purification by column chromatography.

**Scheme 2:**
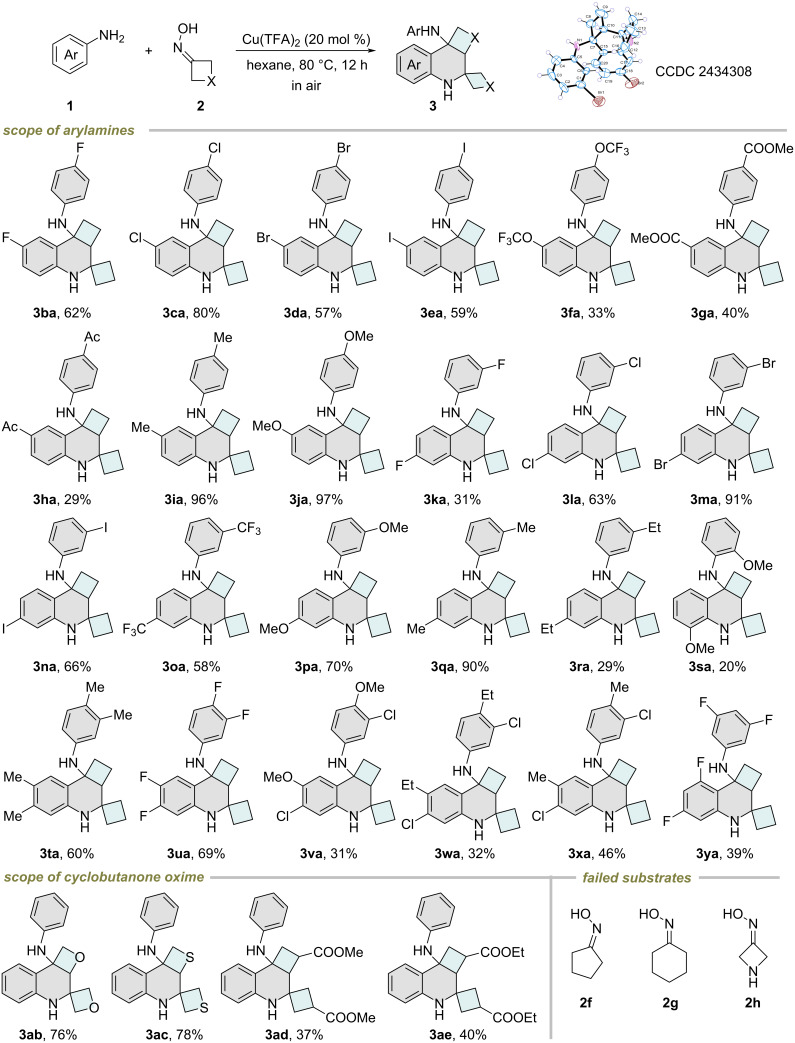
Substrate scope. General reaction conditions: aniline **1** (0.2 mmol), **2** (0.4 mmol), and Cu(TFA)_2_ (0.04 mmol) in hexane (2.0 mmol) under air atmosphere, 12 h, 80 °C. Yields refer to isolated yields.

Having established the optimal reaction conditions, we proceeded to investigate the generality of this Cu-catalyzed system. Initially, a series of anilines bearing diverse substituents was examined, and the results are summarized in Scheme 2. When copper(II) trifluoroacetate was employed as the catalyst, *para*-halogen-substituted anilines **1b**–**e** demonstrated excellent compatibility with the protocol, affording the desired products **3ba**–**ea** in good yields. However, the introduction of strong electron-withdrawing groups, such as trifluoromethoxy, ester, and acetyl, at the *para*-position of the benzene ring (**1f**–**h**) led to a noticeable decrease in the yields of the corresponding STHQs **3fa**–**ha**. In contrast, electron-donating groups, including 4-methylaniline (**1i**) and 4-methoxyaniline (**1j**), were well tolerated, delivering the expected products **3ia** and **3ja** in good yields. Additionally, a variety of *meta*-substituted anilines (**3ka**–**ra**) proved to be suitable substrates for this transformation. However, due to steric hindrance, *ortho*-substituted aniline **1s** exhibited significantly lower reactivity, resulting in diminished yields of product **3sa**. Notably, disubstituted anilines were also compatible with the protocol, furnishing the desired products **3ta**–**ya** in moderate to good yields.

Subsequently, we investigated the scope of cyclobutanones and their analogues in the domino cyclization to access structurally diverse spirotetrahydroquinoline derivatives (Scheme 2). Heterocyclic analogues incorporating oxygen or sulfur atoms within the four-membered ring proved to be compatible substrates, affording cyclo-O/S-containing STHQ derivatives **3ab** and **3ac** in good yields. Additionally, ester-functionalized cyclobutanones exhibited smooth reactivity with aniline, enabling the synthesis of substituted STHQ motifs **3ad** and **3ae** in satisfactory yields. Notably, when cyclopentanone oxime (**2f**), cyclohexanone oxime (**2g**), or azetidinone oxime (**2h**) were employed as alternative substrates to cyclobutanone oxime, the corresponding spirotetrahydroquinoline products were not observed.

**Scheme 3:**
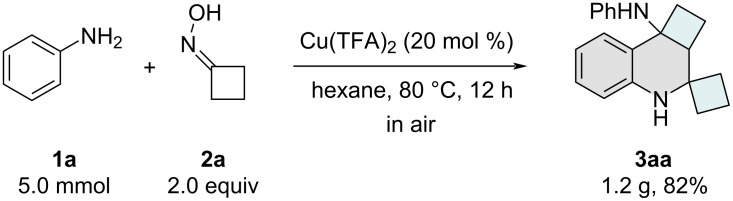
Scale-up reaction.^a^

To showcase the practical utility of our Cu-catalyzed spirotetrahydroquinoline formation process, we conducted a 5.0 mmol scale reaction and obtained the target product **3aa** in 82% yield (Scheme 3).

**Scheme 4:**
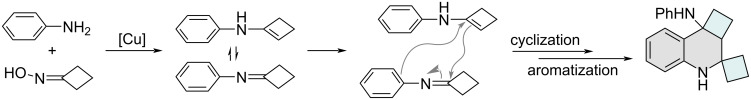
Proposed mechanism.

Based on previous reports, a plausible mechanism was proposed. In the presence of a copper catalyst, aniline reacts with cyclobutanone oxime to form an imine intermediate, which undergoes isomerization to generate an enamine intermediate. Subsequently, an intermolecular cyclization occurs between the enamine and imine intermediates, ultimately yielding the final target product through an aromatization process (Scheme 4).

## Conclusion

In summary, we have developed an efficient and practical copper-catalyzed method for the synthesis of spirotetrahydroquinoline (STHQ) derivatives via the reaction of anilines with cyclobutanone oxime. This protocol offers a straightforward approach to constructing structurally diverse STHQ scaffolds under mild conditions, with broad substrate scope and high functional group tolerance. The optimized reaction conditions, utilizing copper(II) trifluoroacetate as the catalyst and hexane as the solvent, enabled the synthesis of the target products in good to excellent yields. Mechanistic studies suggest a catalytic cycle involving the formation of imine and enamine intermediates, followed by intermolecular cyclization and aromatization. The scalability of this method was demonstrated through a gram-scale reaction, highlighting its potential for practical applications in medicinal chemistry and drug discovery.

## Supporting Information

File 1 Experimental procedures, characterization data for all new compounds, and NMR spectra of products.

## Data Availability

All data that supports the findings of this study is available in the published article and/or the supporting information of this article.
