# Supplementary material for: Copper-catalyzed domino cyclization of anilines and cyclobutanone oxime: a scalable and versatile route to spirotetrahydroquinoline derivatives
Source: Beilstein J Org Chem. 2025 Apr 9;21:749–54. doi: 10.3762/bjoc.21.58 (PMC11995718; doi:10.3762/bjoc.21.58)
Supplement: File 1 — Experimental procedures, characterization data for all new compounds, and NMR spectra of products. [file Beilstein_J_Org_Chem-21-749-s001.pdf]

BEILSTEIN JOURNAL OF ORGANIC CHEMISTRY

Supporting Information

for

Copper-catalyzed domino cyclization of anilines and cyclobutanone oxime: a scalable and versatile route to spirotetrahydroquinoline derivatives

Qingqing Jiang, Xinyi Lei, Pan Gao and Yu Yuan

Beilstein J. Org. Chem. **2025,** *21,* 749–754. doi:10.3762/bjoc.21.58

Experimental procedures, characterization data for all new compounds, and NMR spectra of products

License and Terms: This is a supporting information file under the terms of the Creative Commons Attribution License (https://creativecommons.org/licenses/by/4.0). Please note that the reuse, redistribution and reproduction in particular requires that the author(s) and source are credited and that individual graphics may be subject to special legal provisions.
The license is subject to the *Beilstein Journal of Organic Chemistry* terms and conditions: (https://www.beilstein-journals.org/bjoc/terms)

Table of contents

1. General information..S2

2. The synthesis of cyclobutanone oxime[1]..S2

3. Reaction and method of cyclobutanone oxime with aniline S3

4. Gram scale reaction S4

5. Characterization data for spirotetrahydroquinoline derivatives S5

6. References S37

7. ^{1}H and ^{13}C NMR spectra S38

1. General information

All new compounds were fully characterized. All reactions and manipulations involving air-sensitive compounds were performed using standard Schlenk techniques. Anhydrous MeCN was purchased from Annaiji Chemical and was used as received. ^{1}H and ^{13}C NMR spectra were recorded on an Agilent DD2 400 MHz spectrometer. The chemical shifts in ^{1}H NMR spectra were recorded relative to $CDCl_3$ (δ 7.26). The chemical shifts in ^{13}C NMR spectra were recorded relative to $CDCl_3$ (δ 77.0). The high-resolution mass spectral (HRMS) data were obtained on a quadrupole-type Bruker Dalton MAXIS (APCI). Gas chromatography analyses were conducted with a Shimadzu GC-2014 equipped with ULBON HR-1 glass capillary column (Shinwa Chemical Industries) and an FID detector. Unless otherwise noted, materials obtained from commercial suppliers were used without further purification.

2. The synthesis of cyclobutanone oxime[1]

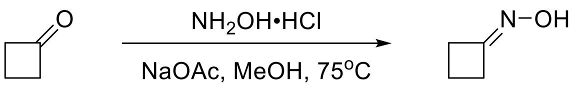

To a mixture of hydroxylamine hydrochloride (1.2 equiv), sodium acetate (2.2 equiv), methanol (100mL) in a 250-mL two-necked flask was added cyclobutanone (1.0 equiv) and the mixture was stirred at 75 °C for 12 h. The reaction mixture was cooled to room temperature and then methanol

was removed under vacuum and the resulting mixture was extracted with diethyl ether. The organic layer was washed with water and dried over $MgSO_4$. The solvent was removed under reduced pressure and the crude material was subjected to column chromatography to afford cyclobutanone oxime in 95% yield.[1]

3. Reaction and method of cyclobutanone oxime with aniline

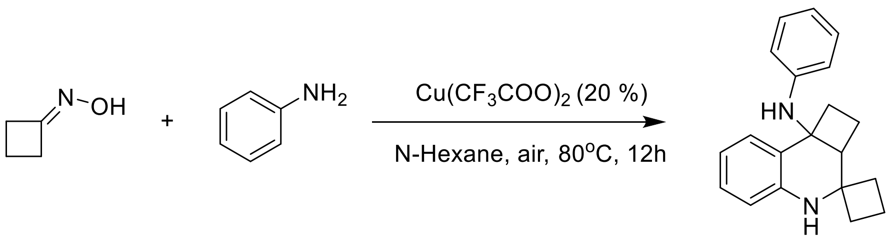

1a **2a** **3aa**, 92%

A dried straight reaction tube was charged with **1** (0.4 mmol), **2** (0.2 mmol), and $Cu(CF_3COO)_2$ (11.58 mg, 20 mol %), then dried *n*-hexane (2 mL) was added by using a syringe. The reaction mixture was stirred at 80 °C for 12 h. After quenching the reaction with aqueous NH_4Cl (2 mL), the crude product was extracted with ethyl acetate (3 × 10 mL). The combined organic phases were dried over anhydrous Na_2SO_4 and concentrated under vacuum. The residue was purified by flash chromatography on neutral alumina to give the desired product **3**.

4. Gram scale reaction

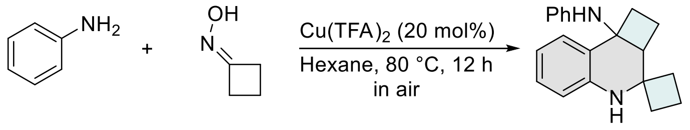

1a 5.0 mmol   **2a** 2.0 equiv.   **3aa** 1.2 g, 82%

A dried two-necked flask was charged with **1a** (10 mmol), **2a** (5 mmol), and $Cu(CF_3COO)_2$ (145 mg, 20 mol %), then dried *n*-hexane (100 mL) was added by using a syringe. The reaction mixture was stirred at 80 °C for 12 h. After quenching the reaction with aqueous NH_4Cl (20 mL), the crude product was extracted with ethyl acetate (3 × 10 mL). The combined organic phases were dried over anhydrous Na_2SO_4 and concentrated under vacuum. The residue was purified by flash chromatography on neutral alumina to give the desired product **3aa** (yellow solid, 1.2 g, 82%).

5. Characterization data for spirotetrahydroquinoline derivatives

(8b'*S*)-*N*-Phenyl-2',2a'-dihydro-1'*H*-spiro[cyclobutane-1,3'-cyclobuta[*c*]quinolin]-8b'(4'*H*)-amine (3aa)[2]

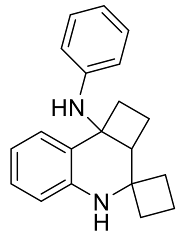

3aa, 92%

The general procedure was applied to aniline (0.2 mmol), cyclobutanone oxime (0.4 mmol), $Cu(CF_3COO)_2$ (11.58 mg, 0.04 mmol), *n*-hexane (2 mL) at 80 °C for 12 h. The crude product was purified by column chromatography on neutral alumina (EtOAc/PE 1:60) to afford the title compound as a yellow solid (27 mg, 92% yield). ^{1}H NMR (400 MHz, $CDCl_3$) δ = 7.32 (dd, *J* = 7.6, 1.5 Hz, 1H), 7.05 (td, *J* = 7.3, 1.1 Hz, 3H), 6.71 (td, *J* = 7.5, 1.2 Hz, 1H), 6.65 (td, *J* = 7.7, 1.1 Hz, 2H), 6.48 (dd, *J* = 8.7, 1.1 Hz, 2H), 4.05 (s, 2H), 2.99 (t, *J* = 8.5 Hz, 1H), 2.39 – 2.30 (m, 1H), 2.20 (dddd, *J* = 11.5, 9.6, 4.6, 1.0 Hz, 1H), 2.05 – 1.83 (m, 5H), 1.77 – 1.68 (m, 1H), 1.68 – 1.58 (m, 2H). ^{13}C NMR (101 MHz, $CDCl_3$) δ = 145.77, 142.98, 128.79, 127.63, 127.36, 126.94, 118.92, 117.54, 115.37, 56.50, 54.77, 50.45, 37.83, 37.52, 33.84, 15.49, 12.66.

7'-Fluoro-*N*-(4-fluorophenyl)-2',2a'-dihydro-1'*H*-spiro[cyclobutane-1,3'-cyclobuta[*c*]quinolin]-8b'(4'*H*)-amine (3ba)[2]

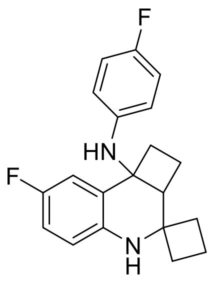

3ba, 62%

The general procedure was applied to 4-fluoroaniline (0.2 mmol), cyclobutanone oxime (0.4 mmol), $Cu(CF_3COO)_2$ (11.58 mg, 0.04 mmol), *n*-hexane (2 mL) at 80 °C for 12 h. The crude product was purified by column chromatography on neutral alumina (EtOAc/PE 1:60) to afford the title compound as a yellow liquid (21 mg, 62% yield). ^{1}H NMR (400 MHz, $CDCl_3$) δ = 7.05 (dd, J = 9.7, 3.0 Hz, 1H), 6.80 – 6.73 (m, 3H), 6.58 (dd, J = 8.7, 4.7 Hz, 1H), 6.41 – 6.35 (m, 2H), 3.92 (s, 2H), 2.88 – 2.78 (m, 1H), 2.24 – 2.13 (m, 2H), 2.06 – 1.88 (m, 3H), 1.83 – 1.72 (m, 3H), 1.61 (dtd, J = 17.2, 8.9, 8.4, 3.0 Hz, 2H).13C NMR (101 MHz, $CDCl_3$) δ 157.96, 155.61, 141.70, 139.28, 129.12, 116.77, 116.32, 115.40, 114.43, 112.96, 57.05, 55.08, 50.23, 37.63, 37.49, 33.56, 15.47, 12.63.

7'-Chloro-*N*-(4-chlorophenyl)-2',2a'-dihydro-1'*H*-spiro[cyclobutane-1,3'-cyclobuta[*c*]quinolin]-8b'(4'*H*)-amine (3ca)[2]

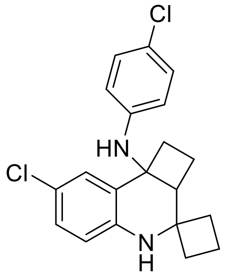

3ca, 80%

The general procedure was applied to 4-chloroaniline (0.2 mmol), cyclobutanone oxime (0.4 mmol), $Cu(CF_3COO)_2$ (11.58 mg, 0.04 mmol), *n*-hexane (2 mL) at 80 °C for 12 h. The crude product was purified by column chromatography on neutral alumina (EtOAc/PE 1:60) to afford the title compound as a yellow solid (29 mg, 80% yield). ^{1}H NMR (400 MHz, $CDCl_3$) δ = 7.24 (d, *J* = 2.5 Hz, 1H), 7.00 (dd, *J* = 8.7, 2.1 Hz, 3H), 6.57 (d, *J* = 8.5 Hz, 1H), 6.40 – 6.33 (m, 2H), 4.21 (s, 1H), 4.01 (s, 1H), 2.86 (t, *J* = 8.5 Hz, 1H), 2.28 – 2.15 (m, 2H), 2.00 (ddt, *J* = 14.2, 11.5, 4.4 Hz, 2H), 1.95 – 1.81 (m, 3H), 1.78 – 1.70 (m, 1H), 1.60 (dtd, *J* = 14.2, 6.1, 5.5, 3.0 Hz, 2H).^{13}C NMR (101 MHz, $CDCl_3$) δ = 143.93, 141.65, 128.70, 128.52, 127.55, 126.46, 123.45, 122.55, 116.63, 116.43, 56.41, 54.83, 50.59, 37.98, 37.73, 33.73, 15.53, 12.60.

7'-Chloro-*N*-(4-chlorophenyl)-2',2a'-dihydro-1'*H*-spiro[cyclobutane-1,3'-cyclobuta[*c*]quinolin]-8b'(4'*H*)-amine (3da)[2]

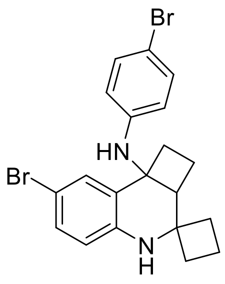

3da, 57%

The general procedure was applied to 4-bromoaniline (0.2 mmol), cyclobutanone oxime (0.4 mmol), $Cu(CF_3COO)_2$ (11.58 mg, 0.04 mmol), *n*-hexane (2 mL) at 80 °C for 12 h. The crude product was purified by column chromatography on neutral alumina (EtOAc/PE 1:60) to afford the title compound as a yellow solid (26 mg, 57% yield). ^{1}H NMR (400 MHz, $CDCl_3$) δ = 7.36 (d, *J* = 2.3 Hz, 1H), 7.12 (dd, *J* = 8.7, 3.4 Hz, 3H), 6.52 (d, *J* = 8.5 Hz, 1H), 6.34 – 6.28 (m, 2H), 4.22 (s, 1H), 4.02 (s, 1H), 2.86 (t, *J* = 8.5 Hz, 1H), 2.28 – 2.15 (m, 2H), 2.04 – 1.95 (m, 2H), 1.94 – 1.81 (m, 3H), 1.78 – 1.69 (m, 1H), 1.63 – 1.55 (m, 2H). ^{13}C NMR (101 MHz, $CDCl_3$) δ = 144.31, 142.07, 131.57, 130.39, 129.33, 128.90, 117.04, 116.86, 110.62, 109.69,56.30, 54.76, 50.61, 38.03, 37.74, 33.74, 15.54, 12.59.

7'-Iodo-N-(4-iodophenyl)-2',2a'-dihydro-1'*H*-spiro[cyclobutane-1,3'-cyclobuta[*c*]quinolin]-8b'(4'*H*)-amine (3ea)[2]

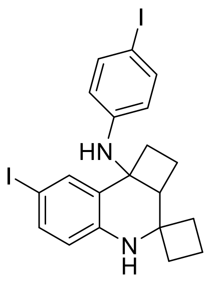

3ea, 59%

The general procedure was applied to 4-iodoaniline (0.2 mmol), cyclobutanone oxime (0.4 mmol), $Cu(CF_3COO)_2$ (11.58 mg, 0.04 mmol), *n*-hexane (2 mL) at 80 °C for 12 h. The crude product was purified by column chromatography on neutral alumina (EtOAc/PE 1:60) to afford the title compound as a yellow solid (32 mg, 59% yield). ^1H NMR (600 MHz, DMSO) δ = 7.25 (d, *J* = 8.2 Hz, 2H), 6.60 (d, *J* = 8.4 Hz, 1H), 6.51 (s, 1H), 6.35 (s, 1H), 6.24 (d, *J* = 8.3 Hz, 2H), 2.73 (t, *J* = 8.2 Hz, 1H), 2.47 (d, *J* = 9.4 Hz, 1H), 2.05 (q, *J* = 9.9 Hz, 1H), 1.92 (dt, *J* = 19.7, 8.9 Hz, 4H), 1.83 (t, *J* = 10.3 Hz, 1H), 1.71 (q, *J* = 10.2 Hz, 1H), 1.54 (q, *J* = 9.4 Hz, 1H), 1.44 (q, *J* = 8.8, 8.4 Hz, 1H). ^{13}C NMR (151 MHz, DMSO) δ 145.67, 143.69, 136.54, 135.20, 133.96, 128.71, 117.51, 116.86, 78.01, 76.98, 54.74, 53.83, 50.83, 37.96, 36.14, 32.68, 15.45, 12.03.

7'-(Trifluoromethoxy)-*N*-(4-(trifluoromethoxy)phenyl)-2',2a'-dihydro-1'*H*-spiro[cyclobutane-1,3'-cyclobuta[*c*]quinolin]-8b'(4'*H*)-amine (3fa)

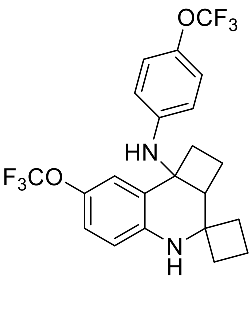

3fa, 33%

The general procedure was applied to 4-(trifluoromethoxy)aniline (0.2 mmol), cyclobutanone oxime (0.4 mmol), $Cu(CF_3COO)_2$ (11.58 mg, 0.04 mmol), *n*-hexane (2 mL) at 80 °C for 12 h. The crude product was purified by column chromatography on neutral alumina (EtOAc/PE 1:20) to afford the title compound as a colorless liquid (15 mg, 33% yield). ^{1}H NMR (400 MHz, $CDCl_3$) δ = 7.13 (dd, *J* = 2.8, 1.0 Hz, 1H), 6.95 – 6.85 (m, 3H), 6.61 (d, *J* = 8.6 Hz, 1H), 6.39 – 6.34 (m, 2H), 4.11 (s, 2H), 2.92 – 2.85 (m, 1H), 2.31 – 2.17 (m, 2H), 2.06 – 1.96 (m, 2H), 1.96 – 1.82 (m, 3H), 1.79 – 1.70 (m, 1H), 1.67 – 1.57 (m, 2H). ^{13}C NMR (101 MHz, $CDCl_3$) δ = 144.02, 141.85, 141.38, 140.93, 127.83, 121.85, 120.79, 119.83, 115.94, 115.71, 56.52, 54.86, 50.60, 37.85, 37.71, 33.69, 15.46, 12.59.

Methyl 8b'-((4-(methoxycarbonyl)phenyl)amino)-2',2a',4',8b'-tetra-hydro-1'*H*-spiro[cyclobutane-1,3'-cyclobuta[*c*]quinoline]-7'-carboxylate (3ga)[2]

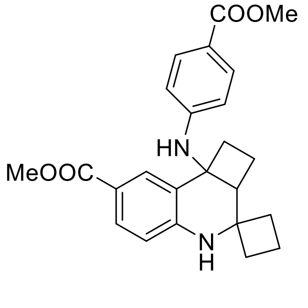

3ga, 40%

The general procedure was applied to methyl 4-aminobenzoate (0.2 mmol), cyclobutanone oxime (0.4 mmol), $Cu(CF_3COO)_2$ (11.58 mg, 0.04 mmol), *n*-hexane (2 mL) at 80 °C for 12 h. The crude product was purified by column chromatography on neutral alumina (EtOAc/PE 1:10) to afford the title compound as a white solid (16 mg, 40% yield). ^{1}H NMR (400 MHz, DMSO) δ = 7.63 (d, *J* = 2.1 Hz, 1H), 7.50 (dd, *J* = 14.9, 8.6 Hz, 3H), 7.20 (s, 1H), 7.09 (s, 1H), 6.69 (d, *J* = 8.6 Hz, 1H), 6.31 (d, *J* = 8.4 Hz, 2H), 3.64 (d, *J* = 7.1 Hz, 6H), 2.79 (t, *J* = 8.9 Hz, 1H), 2.55 (q, *J* = 10.6, 10.2 Hz, 1H), 2.12 – 1.96 (m, 2H), 1.94 – 1.80 (m, 4H), 1.62 (d, *J* = 7.4 Hz, 1H), 1.51 (q, *J* = 9.5 Hz, 1H), 1.31 (p, *J* = 10.0 Hz, 1H). ^{13}C NMR (101 MHz, DMSO) δ = 166.66, 166.64, 150.54, 148.54, 131.50, 130.60, 129.32, 123.69, 117.55, 116.56, 114.24, 113.52, 54.81, 53.92, 51.61, 51.29, 39.05, 36.68, 33.44, 15.90, 12.29.

1-(4-((7'-Acetyl-2',2a'-dihydro-1'*H*-spiro[cyclobutane-1,3'-cyclobuta[*c*]quinoline]-8b'(4'*H*)-yl)amino)phenyl)ethan-1-one (3ha)

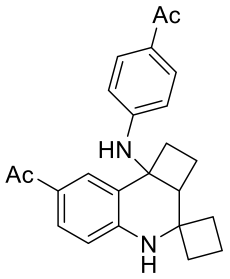

3ha, 29%

The general procedure was applied to 1-(4-aminophenyl)ethan-1-one (0.2 mmol), cyclobutanone oxime (0.4 mmol), $Cu(CF_3COO)_2$ (11.58 mg, 0.04 mmol), *n*-hexane (2 mL) at 80 °C for 12 h. The crude product was purified by column chromatography on neutral alumina (EtOAc/PE 1:10) to afford the title compound as a white solid (11 mg, 29% yield). ^{1}H NMR (400 MHz, DMSO) δ = 7.63 (d, *J* = 2.1 Hz, 1H), 7.57 (dd, *J* = 8.6, 2.1 Hz, 1H), 7.53 (s, 1H), 7.51 (s, 1H), 7.25 (s, 1H), 7.17 (s, 1H), 6.69 (d, *J* = 8.5 Hz, 1H), 6.32 (d, *J* = 8.5 Hz, 2H), 2.81 (t, *J* = 8.9 Hz, 1H), 2.57 (q, *J* = 10.2 Hz, 1H), 2.29 (d, *J* = 5.2 Hz, 6H), 2.10 – 1.81 (m, 6H), 1.69 – 1.59 (m, 1H), 1.58 – 1.49 (m, 1H), 1.37 – 1.25 (m, 1H).^{13}C NMR (101 MHz, DMSO) δ = 195.53, 195.41, 150.61, 148.67, 130.06, 129.32, 128.30, 126.50, 125.47, 123.64, 113.84, 113.34, 54.86, 53.97, 51.22, 39.07, 36.64, 33.44, 26.24, 15.96, 12.32.

7'-Methyl-*N*-(*p*-tolyl)-2',2a'-dihydro-1'*H*-spiro[cyclobutane-1,3'-cyclobuta[*c*]quinolin]-8b'(4'*H*)-amine (3ia)[2]

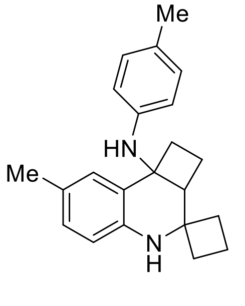

3ia, 96%

The general procedure was applied to *p*-toluidine (0.2 mmol), cyclobutanone oxime (0.4 mmol), $Cu(CF_3COO)_2$ (11.58 mg, 0.04 mmol), *n*-hexane (2 mL) at 80 °C for 12 h. The crude product was purified by column chromatography on neutral alumina (EtOAc/PE 1:60) to afford the title compound as a red liquid (31 mg, 96% yield). ^{1}H NMR (400 MHz, cdcl3) δ = 7.20 (d, *J* = 2.0 Hz, 1H), 6.94 – 6.86 (m, 3H), 6.58 (d, *J* = 8.0 Hz, 1H), 6.47 (d, *J* = 8.4 Hz, 2H), 3.95 (s, 2H), 2.97 (t, *J* = 8.4 Hz, 1H), 2.38 – 2.30 (m, 1H), 2.22 (d, *J* = 4.4 Hz, 6H), 2.17 (dd, *J* = 11.2, 4.8 Hz, 1H), 2.06 – 1.89 (m, 4H), 1.84 (t, *J* = 8.9 Hz, 1H), 1.72 (tt, *J* = 9.5, 4.7 Hz, 1H), 1.69 – 1.58 (m, 2H). ^{13}C NMR (101 MHz, $CDCl_3$) δ = 143.54, 140.58, 129.38, 128.16, 128.07, 127.16, 126.97, 116.02, 115.45, 56.95, 54.89, 49.89, 37.53, 37.44, 33.76, 20.75, 20.42, 15.49, 12.73.

7'-Methoxy-*N*-(4-methoxyphenyl)-2',2a'-dihydro-1'*H*-spiro[cyclobutane-1,3'-cyclobuta[*c*]quinolin]-8b'(4'*H*)-amine (3ja)[2]

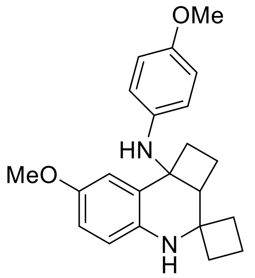

3ja, 97%

The general procedure was applied to 4-methoxyaniline (0.2 mmol), cyclobutanone oxime (0.4 mmol), $Cu(CF_3COO)_2$ (11.58 mg, 0.04 mmol), *n*-hexane (2 mL) at 80 °C for 12 h. The crude product was purified by column chromatography on neutral alumina (EtOAc/PE 1:10) to afford the title compound as a white solid (34 mg, 97% yield). ^{1}H NMR (400 MHz, $CDCl_3$) δ = 7.01 (d, *J* = 2.8 Hz, 1H), 6.70 – 6.65 (m, 3H), 6.59 (d, *J* = 8.4 Hz, 1H), 6.49 (d, *J* = 8.8 Hz, 2H), 3.70 (d, *J* = 6.2 Hz, 6H), 2.87 (t, *J* = 8.4 Hz, 1H), 2.25 (t, *J* = 9.9 Hz, 1H), 2.19 (dd, *J* = 10.0, 4.8 Hz, 1H), 1.99 (dd, *J* = 11.7, 4.1 Hz, 1H), 1.92 (ddd, *J* = 17.3, 8.3, 4.9 Hz, 2H), 1.71 (ddd, *J* = 15.2, 11.4, 7.8 Hz, 3H), 1.64 – 1.50 (m, 2H).^{13}C NMR (101 MHz, $CDCl_3$) δ = 153.89, 152.72, 139.75, 137.05, 129.14, 118.04, 116.48, 114.35, 114.06, 111.52, 57.76, 55.61, 55.55, 54.98, 49.63, 37.48, 37.20, 33.60, 15.35, 12.72.

6'-Fluoro-*N*-(3-fluorophenyl)-2',2a'-dihydro-1'*H*-spiro[cyclobutane-1,3'-cyclobuta[*c*]quinolin]-8b'(4'*H*)-amine (3ka)[2]

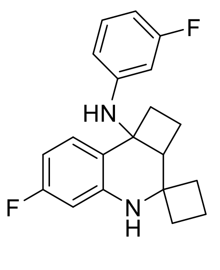

3ka, 31%

The general procedure was applied to 3-fluoroaniline (0.2 mmol), cyclobutanone oxime (0.4 mmol), $Cu(CF_3COO)_2$ (11.58 mg, 0.04 mmol), *n*-hexane (2 mL) at 80 °C for 12 h. The crude product was purified by column chromatography on neutral alumina (EtOAc/PE 1:60) to afford the title compound as a white solid (10 mg, 31% yield). ^1H NMR (600 MHz, $CDCl_3$) δ = 7.19 (dd, *J* = 8.6, 6.4 Hz, 1H), 6.97 (td, *J* = 8.2, 6.8 Hz, 1H), 6.39 (td, *J* = 8.5, 2.5 Hz, 1H), 6.35 – 6.29 (m, 2H), 6.22 (ddd, *J* = 8.2, 2.3, 0.9 Hz, 1H), 6.08 (dt, *J* = 12.0, 2.4 Hz, 1H), 4.33 (s, 1H), 4.08 (s, 1H), 2.93 (t, *J* = 8.7 Hz, 1H), 2.33 – 2.25 (m, 1H), 2.19 – 2.12 (m, 1H), 2.04 (ddt, *J* = 13.0, 8.7, 4.0 Hz, 1H), 1.98 – 1.88 (m, 4H), 1.73 (dtd, *J* = 11.8, 9.6, 4.8 Hz, 1H), 1.63 – 1.57 (m, 2H).^{13}C NMR (151 MHz, $CDCl_3$) δ = 163.32, 161.71, 147.31, 144.42, 129.77, 128.61, 122.03, 111.02, 105.88, 103.97, 101.59, 101.43,56.05, 54.75, 50.82, 38.16, 37.84, 33.91, 15.38, 12.58.

6'-Chloro-*N*-(3-chlorophenyl)-2',2a'-dihydro-1'*H*-spiro[cyclobutane-1,3'-cyclobuta[*c*]quinolin]-8b'(4'*H*)-amine (3la)[2]

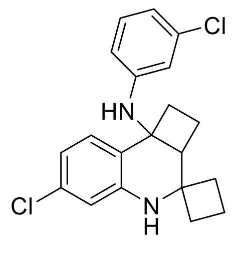

3la, 63%

The general procedure was applied to 3-chloroaniline (0.2 mmol), cyclobutanone oxime (0.4 mmol), $Cu(CF_3COO)_2$ (11.58 mg, 0.04 mmol), *n*-hexane (2 mL) at 80 °C for 12 h. The crude product was purified by column chromatography on neutral alumina (EtOAc/PE 1:60) to afford the title compound as a white solid (23 mg, 63% yield). ^{1}H NMR (400 MHz, $CDCl_3$) δ = 7.19 – 7.13 (m, 1H), 6.94 (t, *J* = 8.1 Hz, 1H), 6.67 – 6.59 (m, 3H), 6.40 (t, *J* = 2.2 Hz, 1H), 6.28 (ddd, *J* = 8.2, 2.3, 0.9 Hz, 1H), 4.30 (s, 1H), 4.07 (s, 1H), 2.89 (t, *J* = 8.5 Hz, 1H), 2.30 – 2.21 (m, 1H), 2.16 (ddd, *J* = 11.5, 10.1, 4.5 Hz, 1H), 2.07 – 1.89 (m, 5H), 1.76 (ddt, *J* = 16.2, 9.8, 4.6 Hz, 1H), 1.67 – 1.58 (m, 2H).^{13}C NMR (101 MHz, $CDCl_3$) δ = 146.53, 144.14, 134.43, 132.92, 129.78, 128.11, 125.05, 118.92, 117.53, 114.88, 114.84, 113.18,55.94, 54.90, 50.92, 38.07, 37.70, 33.80, 15.52, 12.62.

6'-Bromo-*N*-(3-bromophenyl)-2',2a'-dihydro-1'*H*-spiro[cyclobutane-1,3'-cyclobuta[*c*]quinolin]-8b'(4'*H*)-amine (3ma)[2]

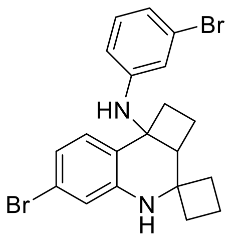

3ma, 91%

The general procedure was applied to 3-bromoaniline (0.2 mmol), cyclobutanone oxime (0.4 mmol), $Cu(CF_3COO)_2$ (11.58 mg, 0.04 mmol), *n*-hexane (2 mL) at 80 °C for 12 h. The crude product was purified by column chromatography on neutral alumina (EtOAc/PE 1:60) to afford the title compound as a white solid (41 mg, 91% yield). ^{1}H NMR (400 MHz, $CDCl_3$) δ 7.08 (d, *J* = 8.7 Hz, 1H), 6.87 (t, *J* = 8.0 Hz, 1H), 6.81 – 6.75 (m, 3H), 6.56 (t, *J* = 2.1 Hz, 1H), 6.(dd, *J* = 7.7, 1.8 Hz, 1H), 4.28 (s, 1H), 4.06 (s, 1H), 2.88 (t, *J* = 8.530 Hz, 1H), 2.29 – 2.14 (m, 2H), 2.06 – 1.89 (m, 5H), 1.77 (ddt, *J* = 11.6, 9.2, 4.7 Hz, 1H), 1.61 (dtd, *J* = 11.8, 8.4, 2.9 Hz, 2H). ^{13}C NMR (101 MHz, $CDCl_3$) δ = 146.63, 144.42, 132.23, 130.08, 128.34, 125.52, 122.70, 121.79, 121.05, 120.43, 117.78, 115.72, 113.49, 55.96, 54.92, 50.93, 38.07, 37.64, 33.78, 15.55, 12.63.

6'-Iodo-*N*-(3-iodophenyl)-2',2a'-dihydro-1'*H*-spiro[cyclobutane-1,3'-cyclobuta[*c*]quinolin]-8b'(4'*H*)-amine (3na)[2]

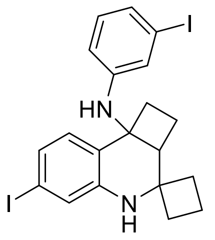

3na, 66%

The general procedure was applied to 3-iodoaniline (0.2 mmol), cyclobutanone oxime (0.4 mmol), $Cu(CF_3COO)_2$ (11.58 mg, 0.04 mmol), *n*-hexane (2 mL) at 80 °C for 12 h. The crude product was purified by column chromatography on neutral alumina (EtOAc/PE 1:60) to afford the title compound as a white solid (36 mg, 66% yield). ^{1}H NMR (400 MHz, $CDCl_3$) δ = 7.03 – 6.91 (m, 4H), 6.78 (s, 1H), 6.73 (t, *J* = 8.0 Hz, 1H), 6.33 (dd, *J* = 8.2, 2.3 Hz, 1H), 4.24 (s, 1H), 4.13 (q, *J* = 7.1 Hz, 1H), 4.05 (s, 1H), 2.87 (t, *J* = 8.5 Hz, 1H), 2.28 – 2.11 (m, 2H), 2.05 (s, 1H), 2.03 – 1.99 (m, 1H), 1.98 – 1.94 (m, 1H), 1.89 (dd, *J* = 10.3, 2.4 Hz, 1H), 1.76 (ddt, *J* = 18.2, 9.2, 4.5 Hz, 1H), 1.61 (dt, *J* = 10.4, 8.4 Hz, 2H), 1.26 (t, *J* = 7.1 Hz, 1H). ^{13}C NMR (101 MHz, $CDCl_3$) δ = 146.55, 144.56, 130.26, 128.49, 127.78, 126.49, 126.28, 123.77, 123.73, 114.03, 94.73, 92.81, 55.96, 54.88, 50.98, 38.09, 37.62, 33.79, 15.59, 12.66.

6'-(Trifluoromethyl)-*N*-(3-(trifluoromethyl)phenyl)-2',2a'-dihydro-1'*H*-spiro[cyclobutane-1,3'-cyclobuta[*c*]quinolin]-8b'(4'*H*)-amine (3oa)[2]

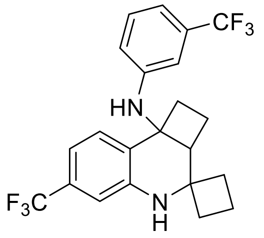

3oa, 58%

The general procedure was applied to 3-(trifluoromethyl)aniline (0.2 mmol), cyclobutanone oxime (0.4 mmol), $Cu(CF_3COO)_2$ (11.58 mg, 0.04 mmol), *n*-hexane (2 mL) at 80 °C for 12 h. The crude product was purified by column chromatography on neutral alumina (EtOAc/PE 1:60) to afford the title compound as a colorless solid (25 mg, 58% yield). ^{1}H NMR (400 MHz, $CDCl_3$) δ = 7.34 (d, *J* = 8.1 Hz, 1H), 7.12 (t, *J* = 8.0 Hz, 1H), 6.95 – 6.87 (m, 3H), 6.64 (s, 1H), 6.52 (dd, *J* = 8.2, 2.4 Hz, 1H), 4.51 (s, 1H), 4.25 (s, 1H), 2.94 (t, *J* = 8.6 Hz, 1H), 2.34 – 2.22 (m, 2H), 2.13 – 2.02 (m, 2H), 2.00 – 1.91 (m, 3H), 1.78 (tdd, *J* = 14.3, 10.2, 5.0 Hz, 1H), 1.64 (dtd, *J* = 17.5, 8.9, 3.1 Hz, 2H).13C NMR (101 MHz, cdcl3) δ 145.40, 143.31, 130.93, 129.72, 129.56, 129.23, 127.35, 125.56, 122.85, 117.66, 115.14, 114.06, 111.88, 111.37, 55.97, 54.76, 51.34, 38.18, 37.70, 33.85, 15.57, 12.53.

6'-Methoxy-*N*-(3-methoxyphenyl)-2',2a'-dihydro-1'*H*-spiro[cyclobutane-1,3'-cyclobuta[*c*]quinolin]-8b'(4'*H*)-amine (3pa)

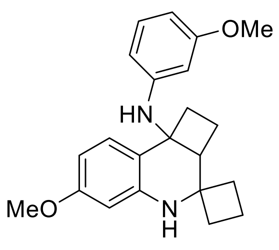

3pa, 70%

The general procedure was applied to 3-methoxyaniline (0.2 mmol), cyclobutanone oxime (0.4 mmol), $Cu(CF_3COO)_2$ (11.58 mg, 0.04 mmol), *n*-hexane (2 mL) at 80 °C for 12 h. The crude product was purified by column chromatography on neutral alumina (EtOAc/PE 1:10) to afford the title compound as a colorless liquid (25 mg, 70% yield). ^{1}H NMR (400 MHz, $CDCl_3$) δ = 7.07 (ddd, *J* = 8.2, 3.9, 1.7 Hz, 1H), 6.82 (dq, *J* = 10.0, 3.9, 3.0 Hz, 1H), 6.17 (ddd, *J* = 6.1, 3.8, 1.8 Hz, 1H), 6.11 – 6.06 (m, 1H), 6.05 – 6.01 (m, 1H), 6.01 – 5.95 (m, 1H), 5.90 (dd, *J* = 4.0, 2.1 Hz, 1H), 3.96 (d, *J* = 84.4 Hz, 2H), 3.61 (dd, *J* = 4.0, 1.8 Hz, 3H), 3.52 – 3.45 (m, 3H), 2.85 (s, 1H), 2.20 (td, *J* = 10.5, 9.9, 3.7 Hz, 1H), 2.08 – 1.97 (m, 1H), 1.93 – 1.69 (m, 5H), 1.62 – 1.53 (m, 1H), 1.51 – 1.42 (m, 2H).^{13}C NMR (101 MHz, $CDCl_3$) δ = 160.24, 159.22, 147.28, 143.95, 129.45, 128.41, 119.75, 108.43, 105.05, 103.04, 101.10, 100.03, 56.24, 55.09, 54.88, 54.67, 50.52, 38.15, 37.72, 33.98, 15.31, 12.64.

6'-Methyl-*N*-(*m*-tolyl)-2',2a'-dihydro-1'*H*-spiro[cyclobutane-1,3'-cyclobuta[*c*]quinolin]-8b'(4'*H*)-amine (3qa)[2]

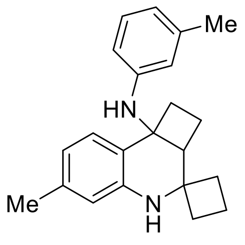

3qa, 90%

The general procedure was applied to *m*-toluidine (0.2 mmol), cyclobutanone oxime (0.4 mmol), $Cu(CF_3COO)_2$ (11.58 mg, 0.04 mmol), *n*-hexane (2 mL) at 80 °C for 12 h. The crude product was purified by column chromatography on neutral alumina (EtOAc/PE 1:60) to afford the title compound as a colorless liquid (29 mg, 90% yield). ^1H NMR (400 MHz, $CDCl_3$) δ = 7.22 (d, *J* = 7.8 Hz, 1H), 6.96 (t, *J* = 7.8 Hz, 1H), 6.55 (d, *J* = 7.8 Hz, 1H), 6.53 – 6.46 (m, 2H), 6.37 (s, 1H), 6.31 (dd, *J* = 8.0, 2.4 Hz, 1H), 4.02 (s, 2H), 3.00 (t, *J* = 8.5 Hz, 1H), 2.36 (dd, *J* = 19.8, 10.0 Hz, 1H), 2.28 (s, 3H), 2.21 (s, 3H), 2.20 – 2.14 (m, 1H), 2.07 – 1.86 (m, 5H), 1.77 – 1.70 (m, 1H), 1.64 (qd, *J* = 9.1, 8.6, 3.0 Hz, 2H). ^{13}C NMR (101 MHz, $CDCl_3$) δ = 145.85, 142.83, 138.45, 137.06, 128.67, 126.82, 125.09, 120.05, 118.43, 116.34, 115.85, 112.34, 56.37, 54.85, 50.30, 37.78, 37.45, 33.87, 21.60, 21.23, 15.48, 12.74.

6'-Ethyl-*N*-(3-ethylphenyl)-2',2a'-dihydro-1'*H*-spiro[cyclobutane-1,3'-cyclobuta[*c*]quinolin]-8b'(4'*H*)-amine (3ra)

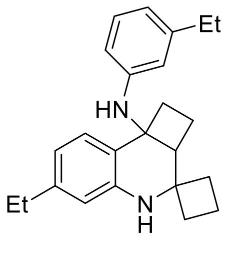

3ra, 29%

The general procedure was applied to 3-ethylaniline (0.2 mmol), cyclobutanone oxime (0.4 mmol), $Cu(CF_3COO)_2$ (11.58 mg, 0.04 mmol), *n*-hexane (2 mL) at 80 °C for 12 h. The crude product was purified by column chromatography on neutral alumina (EtOAc/PE 1:60) to afford the title compound as a yellow liquid (10 mg, 29% yield). ^{1}H NMR (400 MHz, $CDCl_3$) δ = 7.28 (d, *J* = 7.9 Hz, 1H), 7.05 – 6.99 (m, 1H), 6.61 (dd, *J* = 7.9, 1.7 Hz, 1H), 6.55 (d, *J* = 7.8 Hz, 1H), 6.51 (d, *J* = 1.7 Hz, 1H), 6.37 (dd, *J* = 7.8, 1.5 Hz, 2H), 4.07 (d, *J* = 88.6 Hz, 2H), 3.05 (t, *J* = 8.6 Hz, 1H), 2.60 (q, *J* = 7.6 Hz, 2H), 2.52 (q, *J* = 7.6 Hz, 2H), 2.42 (dd, *J* = 20.0, 9.9 Hz, 1H), 2.26 – 2.18 (m, 1H), 2.07 – 1.96 (m, 3H), 1.92 – 1.85 (m, 1H), 1.75 – 1.58 (m, 3H), 1.32 (s, 1H), 1.26 (t, *J* = 7.6 Hz, 3H), 1.16 (t, *J* = 7.6 Hz, 3H).^{13}C NMR (101 MHz, $CDCl_3$) δ = 145.97, 144.77, 143.50, 142.82, 128.67, 127.14, 124.99, 118.82, 117.27, 115.24, 114.52, 112.83, 56.48, 54.66, 50.38, 37.93, 37.42, 34.00, 28.93, 28.58, 15.39, 15.37, 15.27, 12.71.

5'-Methoxy-*N*-(2-methoxyphenyl)-2',2a'-dihydro-1'*H*-spiro[cyclobutane-1,3'-cyclobuta[*c*]quinolin]-8b'(4'*H*)-amine (3sa)

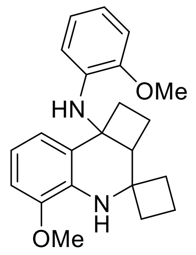

3sa, 20%

The general procedure was applied to 2-methoxyaniline (0.2 mmol), cyclobutanone oxime (0.4 mmol), $Cu(CF_3COO)_2$ (11.58 mg, 0.04 mmol), *n*-hexane (2 mL) at 80 °C for 12 h. The crude product was purified by column chromatography on neutral alumina (EtOAc/PE 1:40) to afford the title compound as a colorless liquid (7 mg, 20% yield). ^{1}H NMR (400 MHz, $CDCl_3$) δ = 6.99 (dd, *J* = 7.4, 1.8 Hz, 1H), 6.82 – 6.75 (m, 1H), 6.72 – 6.57 (m, 4H), 6.33 – 6.25 (m, 1H), 4.91 (s, 1H), 4.74 – 4.60 (m, 1H), 3.90 (d, *J* = 7.0 Hz, 6H), 2.97 (t, *J* = 8.6 Hz, 1H), 2.45 (ddd, *J* = 11.3, 10.0, 8.7 Hz, 1H), 2.22 (ddd, *J* = 11.4, 9.7, 4.1 Hz, 1H), 2.11 – 1.94 (m, 4H), 1.84 (q, *J* = 10.9, 10.0 Hz, 1H), 1.75 – 1.58 (m, 3H).^{13}C NMR (101 MHz, $CDCl_3$) δ = 147.33, 146.61, 135.53, 132.90, 127.51, 120.60, 118.98, 117.32, 116.34, 113.26, 109.31, 107.56, 56.00, 55.53, 55.47, 54.11, 51.07, 38.59, 37.59, 33.82, 15.43, 12.64.

N-(3,4-Dimethylphenyl)-6',7'-dimethyl-2',2a'-dihydro-1'H-spiro[cyclobutane-1,3'-cyclobuta[c]quinolin]-8b'(4'H)-amine (3ta)[2]

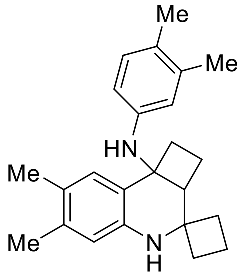

3ta, 60%

The general procedure was applied to 3,4-dimethylaniline (0.2 mmol), cyclobutanone oxime (0.4 mmol), $Cu(CF_3COO)_2$ (11.58 mg, 0.04 mmol), *n*-hexane (2 mL) at 80 °C for 12 h. The crude product was purified by column chromatography on neutral alumina (EtOAc/PE 1:60) to afford the title compound as a yellow liquid (22 mg, 60% yield). ^{1}H NMR (400 MHz, $CDCl_3$) δ = 7.13 (s, 1H), 6.84 (d, *J* = 8.1 Hz, 1H), 6.47 (s, 1H), 6.43 (d, *J* = 2.5 Hz, 1H), 6.30 (dd, *J* = 8.1, 2.5 Hz, 1H), 3.88 (s, 2H), 2.98 (t, *J* = 8.5 Hz, 1H), 2.41 – 2.33 (m, 1H), 2.20 (s, 3H), 2.15 (s, 3H), 2.13 (s, 3H), 2.12 (s, 3H), 2.01 (ddd, *J* = 8.8, 6.4, 3.9 Hz, 2H), 1.95 – 1.88 (m, 2H), 1.87 – 1.80 (m, 1H), 1.78 – 1.64 (m, 2H), 1.64 – 1.56 (m, 2H).^{13}C NMR (101 MHz, $CDCl_3$) δ = 143.99, 140.73, 136.82, 135.60, 129.87, 127.72, 126.95, 125.85, 125.68, 117.86, 116.66, 113.18, 56.71, 54.80, 49.58, 37.48, 37.24, 33.81, 20.01, 19.62, 19.02, 18.70, 15.36, 12.76.

N-(3,4-Difluorophenyl)-6',7'-difluoro-2',2a'-dihydro-1'H-spiro[cyclobutane-1,3'-cyclobuta[c]quinolin]-8b'(4'H)-amine (3ua)

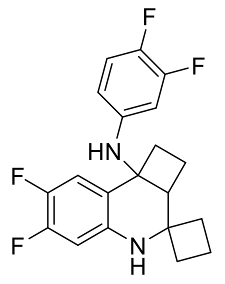

3ua, 69%

The general procedure was applied to 3,4-difluoroaniline (0.2 mmol), cyclobutanone oxime (0.4 mmol), $Cu(CF_3COO)_2$ (11.58 mg, 0.04 mmol), *n*-hexane (2 mL) at 80 °C for 12 h. The crude product was purified by column chromatography on neutral alumina (EtOAc/PE 1:60) to afford the title compound as a yellow solid (25 mg, 69% yield). ^{1}H NMR (400 MHz, $CDCl_3$) δ = 7.06 (dd, J = 11.3, 8.8 Hz, 1H), 6.90 – 6.78 (m, 1H), 6.43 (dd, J = 11.6, 6.8 Hz, 1H), 6.22 – 6.08 (m, 2H), 4.13 (s, 1H), 3.95 (s, 1H), 2.82 (t, J = 8.5 Hz, 1H), 2.22 – 2.11 (m, 2H), 2.07 – 1.91 (m, 3H), 1.88 – 1.82 (m, 2H), 1.79 – 1.72 (m, 1H), 1.64 – 1.57 (m, 2H).^{13}C NMR (101 MHz, $CDCl_3$) δ = 151.57, 151.44, 150.96, 150.82, 149.14, 149.01, 148.52, 148.38, 145.33, 145.20, 144.65, 144.52, 142.96, 142.83, 142.29, 142.26, 142.24, 142.18, 142.16, 139.71, 139.69, 139.63, 139.61, 122.14, 122.10, 122.07, 117.19, 117.17, 117.01, 116.99, 115.09, 115.07, 114.91, 114.89, 110.81, 110.78, 110.76, 110.73, 104.13, 103.92, 103.68, 103.48, 56.35, 54.94, 50.58, 37.87, 33.64, 29.69, 15.31, 12.52.

6'-Chloro-*N*-(3-chloro-4-methoxyphenyl)-7'-methoxy-2',2a'-dihydro-1'*H*-spiro[cyclobutane-1,3'-cyclobuta[*c*]quinolin]-8b'(4'*H*)-amine (3va)

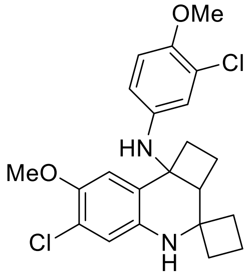

3va, 31%

The general procedure was applied to 3-chloro-4-methoxyaniline (0.2 mmol), cyclobutanone oxime (0.4 mmol), $Cu(CF_3COO)_2$ (11.58 mg, 0.04 mmol), *n*-hexane (2 mL) at 80 °C for 12 h. The crude product was purified by column chromatography on neutral alumina (EtOAc/PE 1:60) to afford the title compound as a yellow liquid (13 mg, 31% yield). ^{1}H NMR (600 MHz, $CDCl_3$) δ = 6.88 (s, 1H), 6.64 (s, 1H), 6.59 (d, *J* = 8.8 Hz, 1H), 6.50 (d, *J* = 2.8 Hz, 1H), 6.23 (dd, *J* = 8.8, 2.8 Hz, 1H), 3.71 (s, 3H), 3.66 (s, 3H), 2.78 – 2.68 (m, 1H), 2.15 – 2.07 (m, 2H), 1.94 – 1.85 (m, 2H), 1.81 (dt, *J* = 12.0, 8.8 Hz, 1H), 1.73 – 1.69 (m, 2H), 1.59 – 1.46 (m, 3H). ^{13}C NMR (151 MHz, $CDCl_3$) δ = 148.40, 147.99, 140.15, 137.60, 126.92, 118.45, 117.10, 115.18, 113.35, 111.09, 57.25, 56.82, 56.71, 55.04, 50.36, 37.82, 37.47, 33.51, 15.35, 12.69.

6'-Chloro-*N*-(3-chloro-4-ethylphenyl)-7'-ethyl-2',2a'-dihydro-1'*H*-spiro[cyclobutane-1,3'-cyclobuta[*c*]quinolin]-8b'(4'*H*)-amine (3wa)[2]

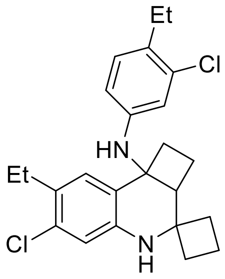

3wa, 32%

The general procedure was applied to 3-chloro-4-ethylaniline (0.2 mmol), cyclobutanone oxime (0.4 mmol), $Cu(CF_3COO)_2$ (11.58 mg, 0.04 mmol), *n*-hexane (2 mL) at 80 °C for 12 h. The crude product was purified by column chromatography on neutral alumina (EtOAc/PE 1:60) to afford the title compound as a red liquid (13 mg, 32% yield). ^{1}H NMR (400 MHz, $CDCl_3$) δ = 7.13 (s, 1H), 6.90 (d, *J* = 8.3 Hz, 1H), 6.67 (s, 1H), 6.49 (d, *J* = 2.5 Hz, 1H), 6.31 (dd, *J* = 8.3, 2.4 Hz, 1H), 4.02 (d, *J* = 70.7 Hz, 2H), 2.90 (t, *J* = 8.5 Hz, 1H), 2.59 (p, *J* = 7.3 Hz, 4H), 2.28 (q, *J* = 10.0 Hz, 1H), 2.16 (td, *J* = 11.1, 10.6, 4.5 Hz, 1H), 2.04 – 1.85 (m, 5H), 1.74 (ddd, *J* = 14.1, 9.2, 4.7 Hz, 1H), 1.67 – 1.57 (m, 2H), 1.13 (dt, *J* = 19.2, 7.5 Hz, 6H). ^{13}C NMR (101 MHz, $CDCl_3$) δ = 144.60, 141.99, 133.72, 132.30, 131.77, 130.53, 129.48, 127.65, 126.19, 116.09, 115.60, 114.26, 56.45, 54.95, 50.46, 37.71, 37.59, 33.69, 26.04, 25.73, 15.44, 14.65, 14.38, 12.68.

6'-Chloro-*N*-(3-chloro-4-methylphenyl)-7'-methyl-2',2a'-dihydro-1'*H*-spiro[cyclobutane-1,3'-cyclobuta[*c*]quinolin]-8b'(4'*H*)-amine (3xa)

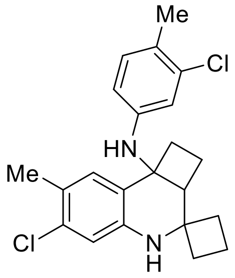

3xa, 46%

The general procedure was applied to 3-chloro-4-methylaniline (0.2 mmol), cyclobutanone oxime (0.4 mmol), $Cu(CF_3COO)_2$ (11.58 mg, 0.04 mmol), *n*-hexane (2 mL) at 80 °C for 12 h. The crude product was purified by column chromatography on neutral alumina (EtOAc/PE 1:60) to afford the title compound as a yellow solid (18 mg, 46% yield). ^{1}H NMR (400 MHz, $CDCl_3$) δ = 7.11 (s, 1H), 6.88 (d, *J* = 8.2 Hz, 1H), 6.67 (s, 1H), 6.48 (d, *J* = 2.4 Hz, 1H), 6.26 (dd, *J* = 8.3, 2.5 Hz, 1H), 4.01 (s, 2H), 2.86 (t, *J* = 8.5 Hz, 1H), 2.19 (d, *J* = 5.3 Hz, 8H), 2.04 – 1.85 (m, 5H), 1.74 (ddt, *J* = 14.0, 9.3, 4.7 Hz, 1H), 1.61 (t, *J* = 8.7 Hz, 2H).^{13}C NMR (101 MHz, $CDCl_3$) δ = 144.58, 142.01, 134.28, 132.88, 130.87, 128.76, 125.96, 125.82, 124.66, 115.77, 115.40, 113.93, 56.26, 54.99, 50.47, 37.79, 37.65, 33.70, 19.17, 18.84, 15.47, 12.65.

_N_-(3,5-Difluorophenyl)-6',8'-difluoro-2',2a'-dihydro-1'_H_-spiro[cyclobutane-1,3'-cyclobuta[_c_]quinolin]-8b'(4'_H_)-amine (3ya)[2]

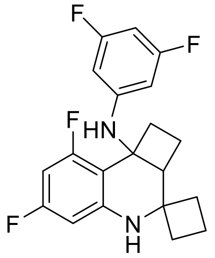

3ya, 39%

The general procedure was applied to 3,5-difluoroaniline (0.2 mmol), cyclobutanone oxime (0.4 mmol), $Cu(CF_3COO)_2$ (11.58 mg, 0.04 mmol), _n_-hexane (2 mL) at 80 °C for 12 h. The crude product was purified by column chromatography on neutral alumina (EtOAc/PE 1:60) to afford the title compound as a white solid (12 mg, 39% yield). ^{1}H NMR (400 MHz, $CDCl_3$) δ = 6.14 – 5.99 (m, 3H), 5.96 – 5.82 (m, 2H), 4.53 (s, 1H), 4.39 (s, 1H), 2.97 – 2.90 (m, 1H), 2.43 – 2.29 (m, 2H), 2.08 (td, _J_ = 7.9, 3.8 Hz, 3H), 1.99 – 1.90 (m, 2H), 1.74 – 1.60 (m, 2H), 1.52 – 1.44 (m, 1H).^{13}C NMR (101 MHz, $CDCl_3$) δ = 164.85, 164.69, 164.08, 163.99, 163.91, 163.84, 162.43, 162.28, 161.64, 161.52, 161.48, 161.37, 147.80, 147.67, 147.54, 145.62, 145.49, 145.39, 105.80, 105.68, 105.65, 97.27, 97.18, 97.06, 96.98, 96.15, 96.12, 95.91, 95.88, 93.55, 93.29, 93.03, 92.59, 92.33, 92.07, 53.93, 53.59, 50.26, 38.93, 35.28, 34.78, 16.44, 12.29.

N-Phenyl-1'H,4'H-spiro[oxetane-3,3'-oxeto[2,3-*c*]quinolin]-8b'(2a'H)-amine (3ab)

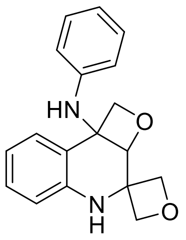

3ab, 76%

The general procedure was applied to aniline (0.2 mmol), oxetan-3-one (0.4 mmol), $Cu(CF_3COO)_2$ (11.58 mg, 0.04 mmol), n-hexane (2 mL) at 80 °C for 12 h. The crude product was purified by column chromatography on neutral alumina (EtOAc/PE 1:5) to afford the title compound as a white solid (22 mg, 76% yield). ^{1}H NMR (400 MHz, DMSO) δ = 7.00 (ddd, J = 19.9, 8.2, 6.8 Hz, 4H), 6.94 – 6.86 (m, 2H), 6.77 (s, 1H), 6.59 (t, J = 7.4 Hz, 1H), 6.52 (t, J = 7.3 Hz, 1H), 6.21 (d, J = 7.6 Hz, 2H), 5.01 (d, J = 2.0 Hz, 1H), 4.75 (d, J = 5.8 Hz, 1H), 4.57 (d, J = 6.5 Hz, 1H), 4.50 (d, J = 6.6 Hz, 1H), 4.36 (d, J = 6.0 Hz, 1H), 4.20 (dd, J = 16.1, 5.9 Hz, 2H).^{13}C NMR (101 MHz, DMSO) δ = 145.94, 143.71, 143.66, 129.19, 128.33, 125.41, 124.37, 118.88, 117.16, 116.35, 116.32, 114.24, 92.78, 82.16, 81.21, 78.21, 56.80, 55.63.

_N_-Phenyl-1'_H_,4'_H_-spiro[thietane-3,3'-thieto[2,3-_c_]quinolin]-8b'(2a'_H_)-amine (3ac)[2]

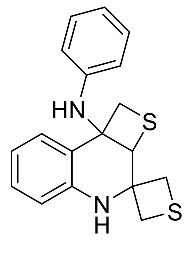

3ac, 78%

The general procedure was applied to aniline (0.2 mmol), thietan-3-one (0.4 mmol), $Cu(CF_3COO)_2$ (11.58 mg, 0.04 mmol), *n*-hexane (2 mL) at 80 °C for 12 h. The crude product was purified by column chromatography on neutral alumina (EtOAc/PE 1:10) to afford the title compound as a white solid (25 mg, 78% yield). ^{1}H NMR (600 MHz, $CDCl_3$) δ = 7.31 (dd, *J* = 7.8, 1.5 Hz, 1H), 7.11 (dd, *J* = 8.8, 7.4 Hz, 3H), 6.80 (td, *J* = 7.5, 1.2 Hz, 1H), 6.73 (t, *J* = 7.4 Hz, 2H), 6.64 (d, *J* = 7.5 Hz, 2H), 4.96 (s, 1H), 4.58 (s, 1H), 4.35 (s, 1H), 3.72 (d, *J* = 9.6 Hz, 1H), 3.14 (d, *J* = 10.3 Hz, 1H), 3.07 (d, *J* = 9.6 Hz, 1H), 2.99 (dd, *J* = 11.7, 2.2 Hz, 3H).^{13}C NMR (151 MHz, $CDCl_3$) δ = 144.63, 141.16, 129.48, 128.95, 127.96, 126.23, 120.77, 119.15, 116.74, 115.55, 61.02, 59.37, 56.41, 40.56, 39.12, 37.71.

Dimethyl 8b'-(phenylamino)-2',2a',4',8b'-tetrahydro-1'*H*-spiro[cyclobutane-1,3'-cyclobuta[*c*]quinoline]-2',3-dicarboxylate (3ad)[2]

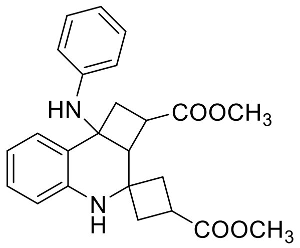

3ad, 37%

The general procedure was applied to aniline (0.2 mmol), methyl 3-oxocyclobutane-1-carboxylate (0.4 mmol), $Cu(CF_3COO)_2$ (11.58 mg, 0.04 mmol), *n*-hexane (2 mL) at 80 °C for 12 h. The crude product was purified by column chromatography on neutral alumina (EtOAc/PE 1:10) to afford the title compound as a colorless liquid (15 mg, 37% yield). ^{1}H NMR (400 MHz, $CDCl_3$) δ = 7.40 (d, *J* = 7.7 Hz, 1H), 7.13 – 7.02 (m, 3H), 6.79 (t, *J* = 7.5 Hz, 1H), 6.70 (dd, *J* = 14.6, 7.6 Hz, 2H), 6.46 (d, *J* = 8.7 Hz, 2H), 4.34 (s, 1H), 4.11 (s, 1H), 3.73 (s, 3H), 3.61 (s, 3H), 3.18 – 3.06 (m, 2H), 2.84 (dt, *J* = 9.1, 6.9 Hz, 1H), 2.69 – 2.56 (m, 2H), 2.35 (dd, *J* = 12.1, 9.6 Hz, 1H), 2.26 – 2.20 (m, 1H), 2.14 (d, *J* = 8.6 Hz, 2H). ^{13}C NMR (101 MHz, $CDCl_3$) δ = 175.94, 175.30, 145.41, 142.39, 128.88, 128.01, 127.71, 126.78, 120.02, 118.35, 116.26, 115.85, 56.53, 54.12, 52.91, 52.11, 51.90, 40.03, 39.80, 35.69, 32.58, 30.56.

Diethyl 8b'-(phenylamino)-2',2a',4',8b'-tetrahydro-1'*H*-spiro[cyclobutane-1,3'-cyclobuta[*c*]quinoline]-2',3-dicarboxylate (3ae)

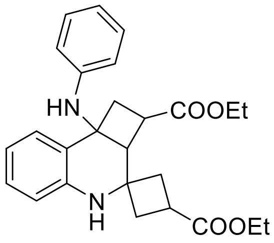

3ae, 40%

The general procedure was applied to aniline (0.2 mmol), 3-oxocyclobutyl propionate (0.4 mmol), $Cu(CF_3COO)_2$ (11.58 mg, 0.04 mmol), *n*-hexane (2 mL) at 80 °C for 12 h. The crude product was purified by column chromatography on neutral alumina (EtOAc/PE 1:10) to afford the title compound as a white solid (17 mg, 40% yield). ^{1}H NMR (400 MHz, $CDCl_3$) δ = 7.37 (d, *J* = 7.8 Hz, 1H), 7.12 – 7.01 (m, 3H), 6.76 (t, *J* = 7.5 Hz, 1H), 6.73 – 6.65 (m, 2H), 6.52 – 6.45 (m, 2H), 4.49 (s, 1H), 4.32 (s, 1H), 4.21 – 4.09 (m, 4H), 3.07 (d, *J* = 7.1 Hz, 1H), 2.87 – 2.79 (m, 1H), 2.75 (q, *J* = 7.6 Hz, 1H), 2.67 (dd, *J* = 11.8, 7.5 Hz, 1H), 2.47 (ddd, *J* = 12.3, 8.8, 3.1 Hz, 1H), 2.38 (dd, *J* = 11.8, 9.3 Hz, 1H), 2.28 – 2.18 (m, 2H), 2.05 (dd, *J* = 12.0, 7.5 Hz, 1H), 1.26 (dd, *J* = 15.5, 6.9 Hz, 7H). ^{13}C NMR (101 MHz, $CDCl_3$) δ = 176.35, 175.56, 145.46, 142.17, 128.93, 127.83, 126.96, 126.93, 119.69, 118.24, 116.10, 115.70, 60.84, 60.79, 55.55, 54.02, 51.48, 40.64, 40.46, 36.12, 32.81, 29.65, 14.23, 14.19.

Single-crystal data of compound 3aa

Datablock jqq01 - ellipsoid plot

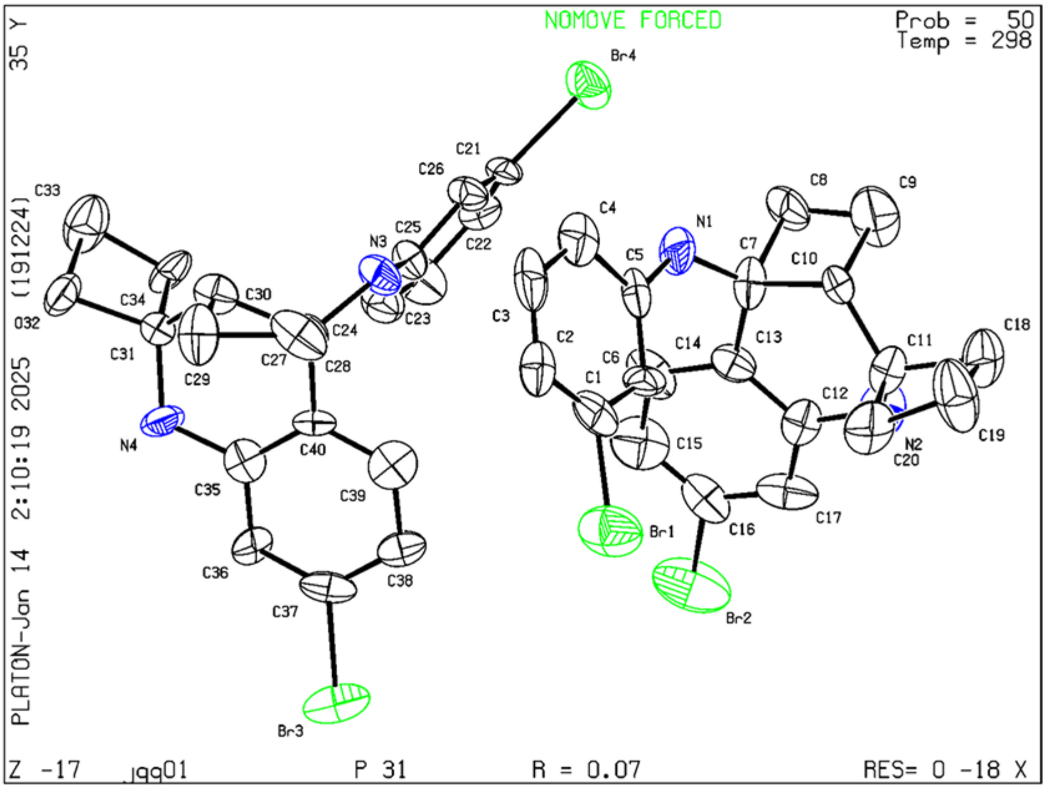

checkCIF/PLATON report

Structure factors have been supplied for datablock(s) jqq01

THIS REPORT IS FOR GUIDANCE ONLY. IF USED AS PART OF A REVIEW PROCEDURE FOR PUBLICATION, IT SHOULD NOT REPLACE THE EXPERTISE OF AN EXPERIENCED CRYSTALLOGRAPHIC REFEREE.

No syntax errors found. CIF dictionary Interpreting this report

Datablock: jqq01

Bond precision:	C-C = 0.0248 A	Wavelength=0.71073	
Cell:	a=10.5820(15)	b=10.5820(15)	c=28.192(4)
	alpha=90	beta=90	gamma=120
Temperature:	298 K		

	Calculated	Reported
Volume	2734.0(9)	2734.0(9)
Space group	P 31	P 31
Hall group	P 31	P 31
Moiety formula	C20 Br2 N2	2(Br2 C20 N2)
Sum formula	C20 Br2 N2	C40 Br4 N4
Mr	428.02	856.08
Dx,g cm-3	1.560	1.560
Z	6	3
Mu (mm-1)	4.448	4.448
F000	1224.0	1224.0
F000'	1221.11	
h,k,lmax	13,13,36	13,13,36
Nref	8446[4223]	6917
Tmin,Tmax	0.566,0.641	0.422,0.746
Tmin'	0.555	

Correction method= # Reported T Limits: Tmin=0.422 Tmax=0.746
AbsCorr = NONE

Data completeness= 1.64/0.82 Theta(max)= 27.568

R(reflections)= 0.0702(3031) wR2(reflections)= 0.1554(6917)

S = 0.953 Npar= 434

```
The following ALERTS were generated. Each ALERT has the format
       test-name_ALERT_alert-type_alert-level.
Click on the hyperlinks for more details of the test.
```

Alert level B

```
PLAT241_ALERT_2_B High   'MainMol' Ueq as Compared to Neighbors of        C9 Check
PLAT341_ALERT_3_B Low Bond Precision on  C-C Bonds ...............    0.02477 Ang.
```

Alert level C

```
PLAT041_ALERT_1_C Calc. and Reported SumFormula    Strings  Differ     Please Check
              Calc: C20 Br2 N2
              Rep.: C40 Br4 N4
PLAT042_ALERT_1_C Calc. and Reported MoietyFormula Strings  Differ     Please Check
              Calc: C20 Br2 N2
              Rep.: 2(Br2 C20 N2)
PLAT057_ALERT_3_C Correction for Absorption Required   RT(exp) ...       1.13 Do !
PLAT241_ALERT_2_C High   'MainMol' Ueq as Compared to Neighbors of        N2 Check
PLAT241_ALERT_2_C High   'MainMol' Ueq as Compared to Neighbors of       C19 Check
PLAT241_ALERT_2_C High   'MainMol' Ueq as Compared to Neighbors of       C33 Check
PLAT242_ALERT_2_C Low    'MainMol' Ueq as Compared to Neighbors of        C8 Check
PLAT242_ALERT_2_C Low    'MainMol' Ueq as Compared to Neighbors of       C10 Check
PLAT242_ALERT_2_C Low    'MainMol' Ueq as Compared to Neighbors of       C16 Check
PLAT906_ALERT_3_C Large K Value in the Analysis of Variance ......      4.669 Check
PLAT906_ALERT_3_C Large K Value in the Analysis of Variance ......      2.391 Check
PLAT911_ALERT_3_C Missing FCF Refl Between Thmin & STh/L=    0.600         62 Report
               -6 11  0,   5  6  1,   0  1  2,   5  6  2,  -6 10  6,  -5 10  8,
               -4 10  9,  -3 10  9,  -8  9 11,  -7  9 11, -11  7 12,  -8  9 12,
               -7  9 12,  -5  9 12,  -7  9 13,  -6  9 13,  -3  9 13, -10  7 14,
               -8  8 14,  -6  9 14,  -9  7 15,  -7  8 15,  -6  8 15,  -5  8 15,
               -8  7 16,  -4  7 16,  -7  8 16,  -6  8 16,  -5  8 16,  -4  8 16,
               -8  7 17,  -7  7 17,  -4  7 17,  -6  8 17,  -5  8 17,  -4  8 17,
               -8  6 18,  -7  7 18,  -6  7 18,  -4  7 18,  -5  8 18,  -4  8 18,
               -6  7 19,  -5  7 19,  -4  7 19,  -3  7 19,  -1  7 19,  -5  8 19,
               -4  8 19,  -5  7 20,  -4  7 20,  -6  6 21,  -5  6 21,  -6  6 22,
               -5  6 22,  -2  6 22,  -6  5 23,  -5  6 23,  -6  5 24,  -5  5 24,
                0  5 25,   0  0 30,
PLAT915_ALERT_3_C No Flack x Check Done: Low Friedel Pair Coverage        66 %
```

Alert level G

```
PLAT002_ALERT_2_G Number of Distance or Angle Restraints on AtSite        16 Note
PLAT040_ALERT_1_G No H-atoms in this Carbon Containing Compound ..     Please Check
PLAT045_ALERT_1_G Calculated and Reported Z Differ by a Factor ...         2 Check
PLAT172_ALERT_4_G The CIF-Embedded .res File Contains DFIX Records         4 Report
PLAT850_ALERT_4_G Check Flack Parameter Exact Value 0.00 with s.u.      0.02 Check
PLAT860_ALERT_3_G Number of Least-Squares Restraints .............         9 Note
PLAT910_ALERT_3_G Missing # of FCF Reflection(s) Below Theta(Min).         3 Note
                0  1  0,  -1  1  1,   0  0  3,
PLAT912_ALERT_4_G Missing # of FCF Reflections Above STh/L=  0.600        21 Note
PLAT933_ALERT_2_G Number of HKL-OMIT Records in Embedded .res File         2 Note
                0  1  2,  -1  1 -2,
PLAT941_ALERT_3_G Average HKL Measurement Multiplicity ...........       2.8 Low
PLAT969_ALERT_5_G The 'Henn et al.' R-Factor-gap value ...........     1.245 Note
```

6. References

[1] Olive, G.; Le Moigne, F.; Mercier, A.; Rockenbauer, A.; Tordo, P. *J. Org. Chem.* **1998**, *63*, 9095.

[2] Chen, C.; Wang, Z.; Wang, S.; Xu, L.; Zeng, X. *Org. Lett.* **2023**, *25*, 4241.

7. ^{1}H and ^{13}C NMR spectra

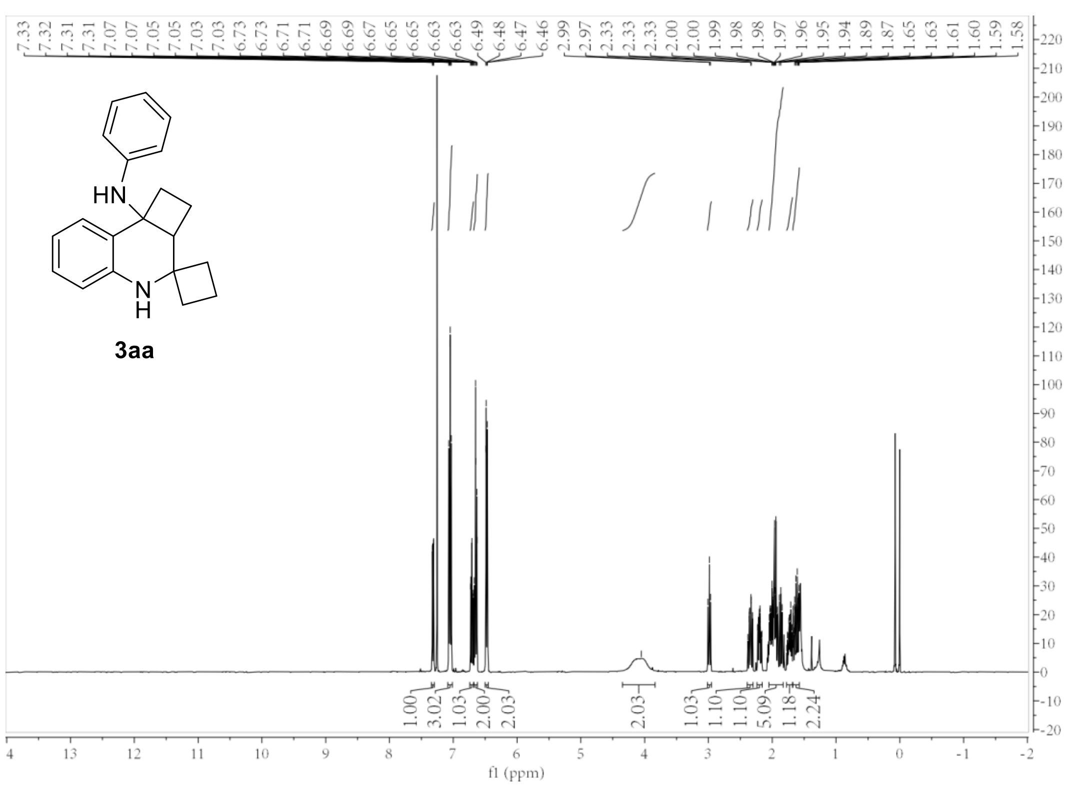

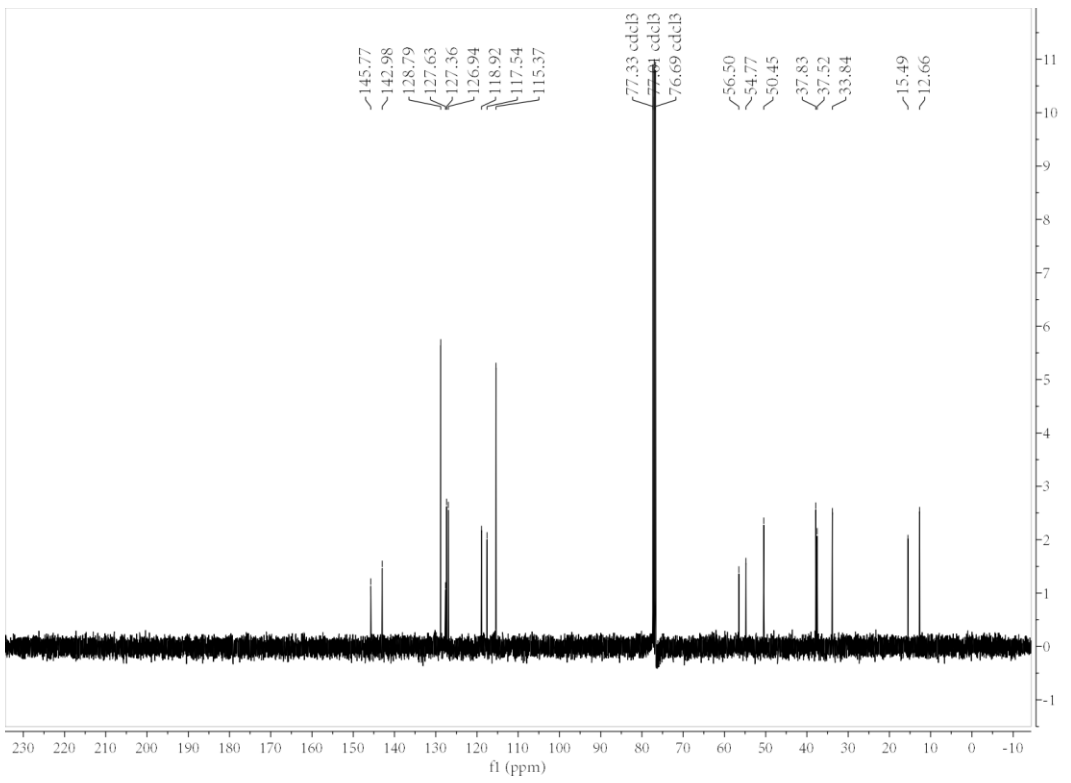

Figure S1. ^{1}H (400 MHz, $CDCl_3$) and ^{13}C (101 MHz, $CDCl_3$) NMR spectra for compound **3aa**

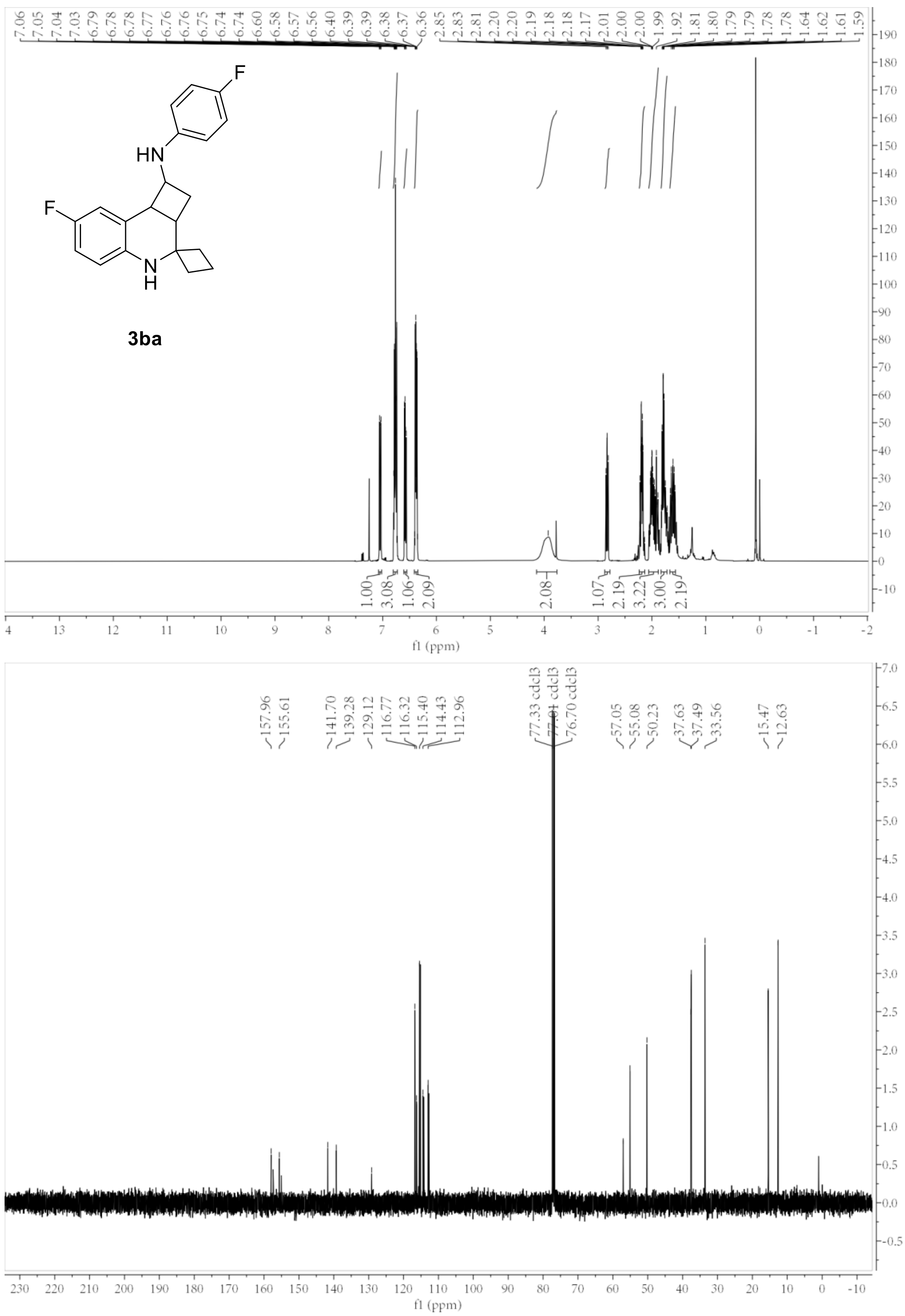

Figure S2. ^{1}H (400 MHz, $CDCl_3$) and ^{13}C (101 MHz, $CDCl_3$) NMR spectra for compound **3ba**

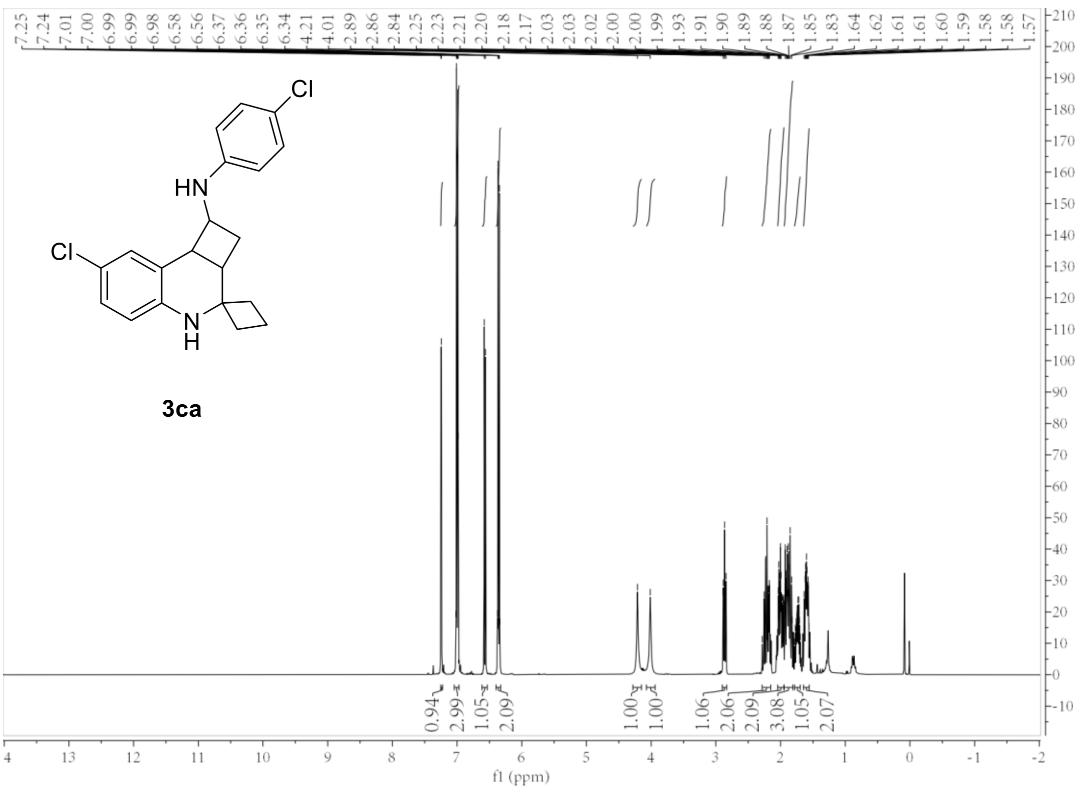

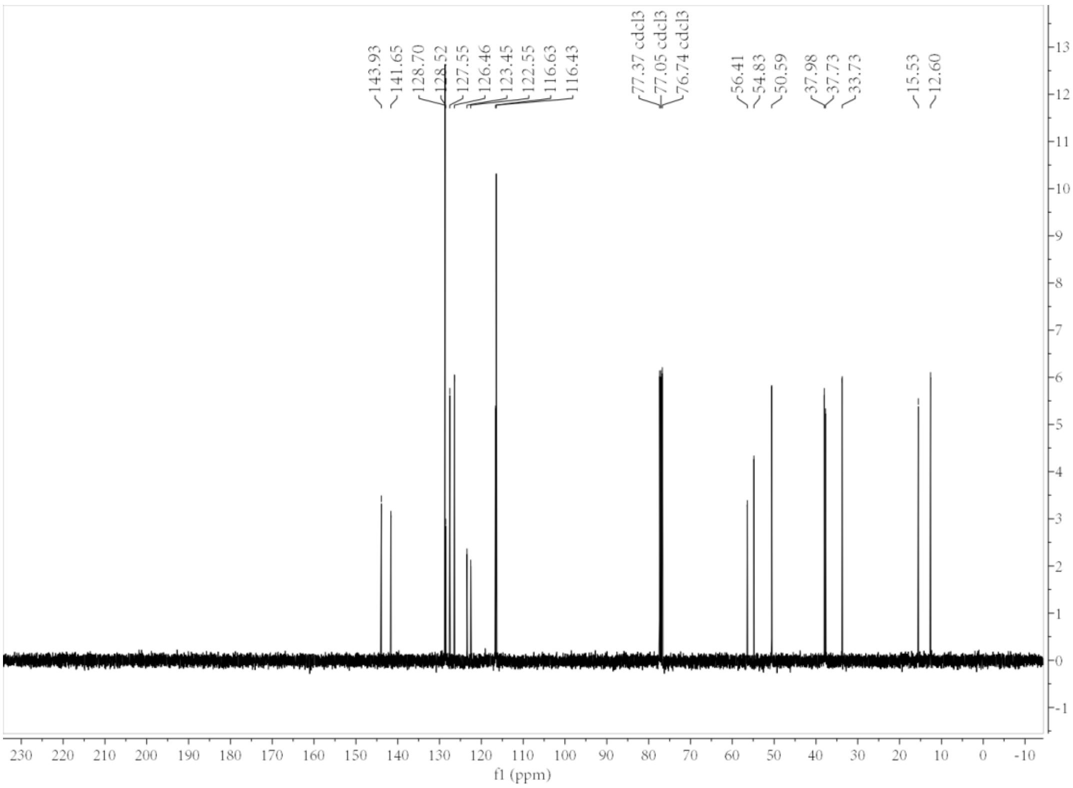

Figure S3. ^{1}H (400 MHz, $CDCl_3$) and ^{13}C (101 MHz, $CDCl_3$) NMR spectra for compound **3ca**

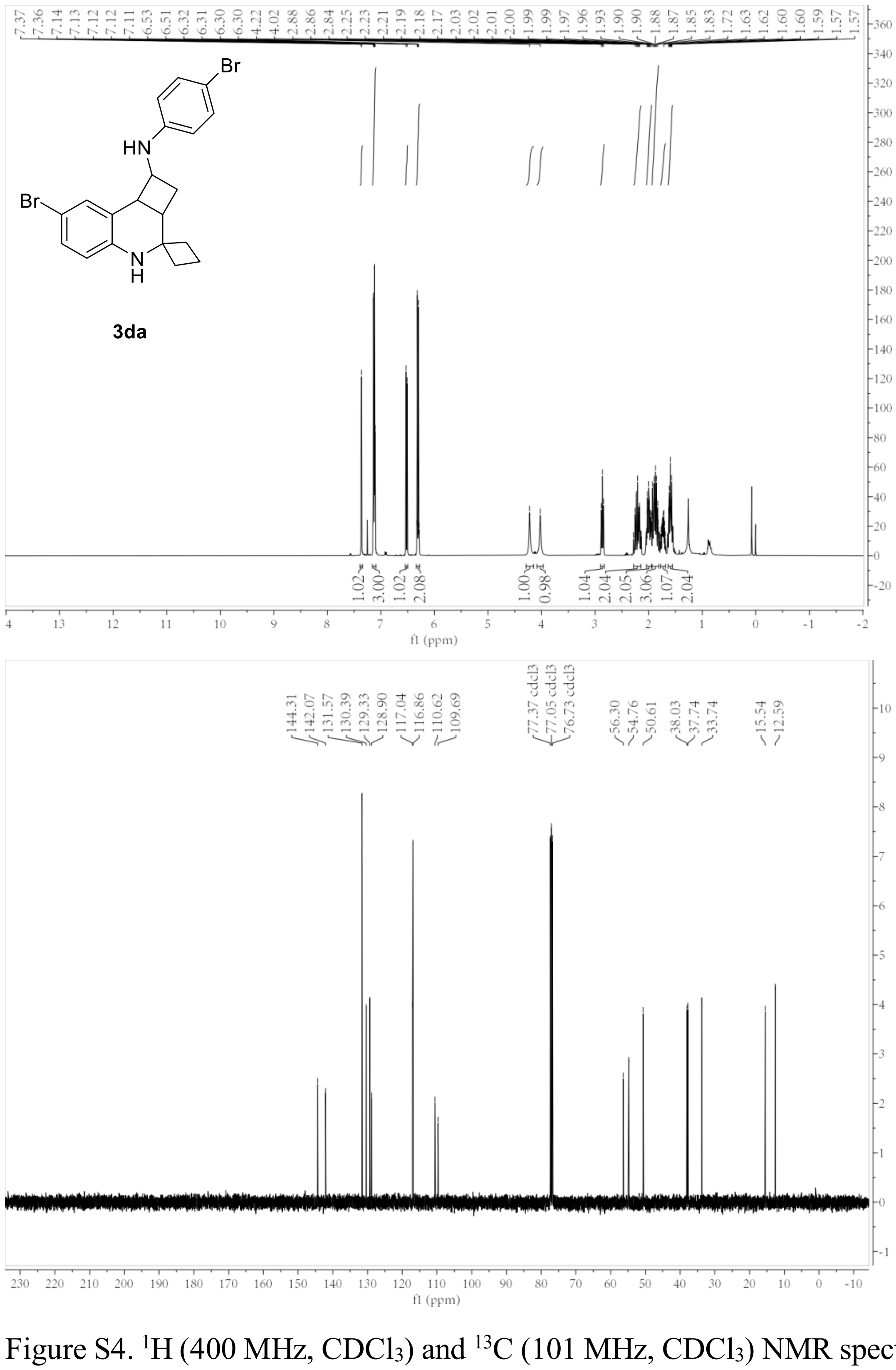

Figure S4. ^{1}H (400 MHz, $CDCl_3$) and ^{13}C (101 MHz, $CDCl_3$) NMR spectra for compound **3da**

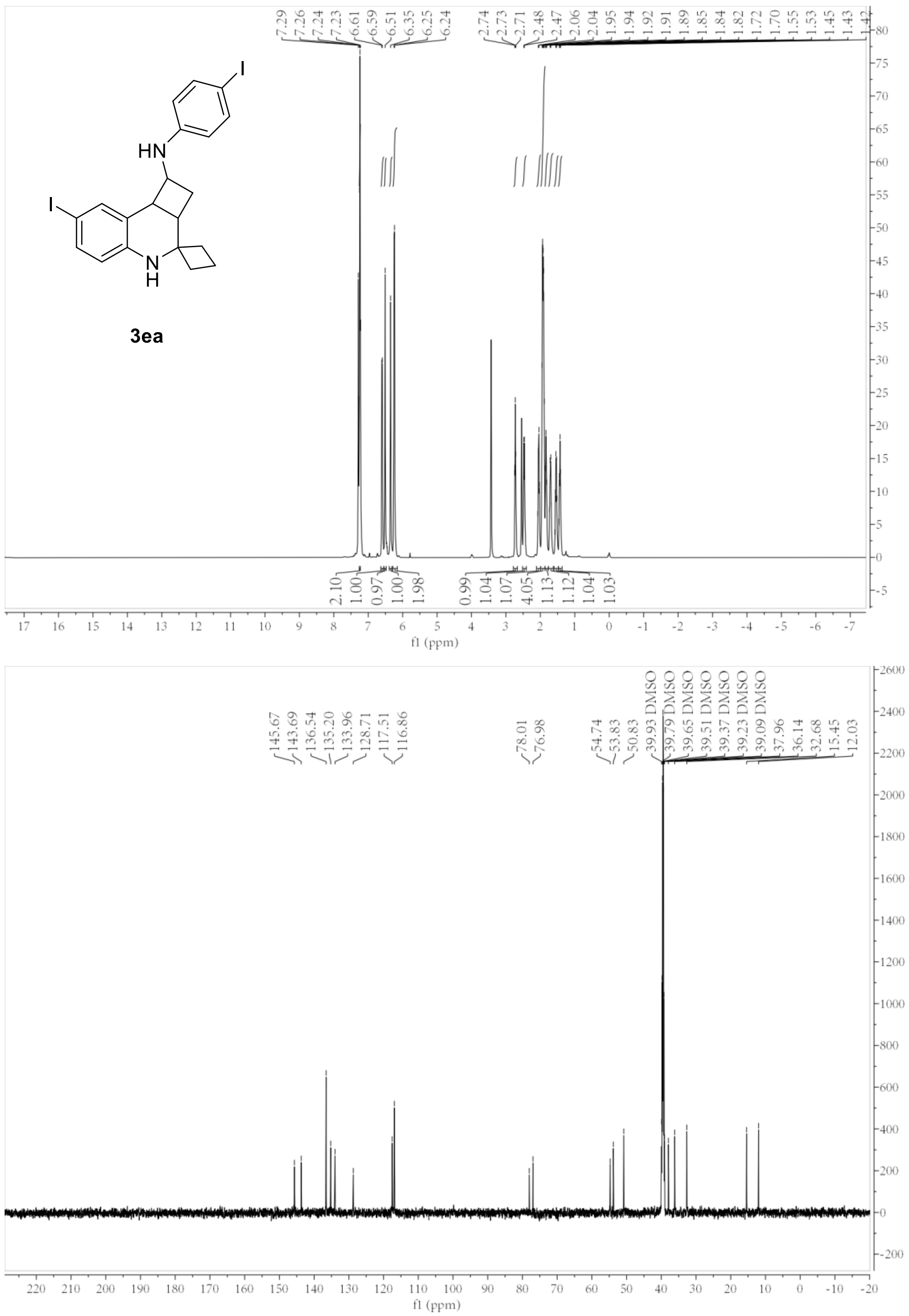

Figure S5. ^{1}H(600 MHz, DMSO) and ^{13}C (151 MHz, DMSO) NMR spectra for compound **3ea**

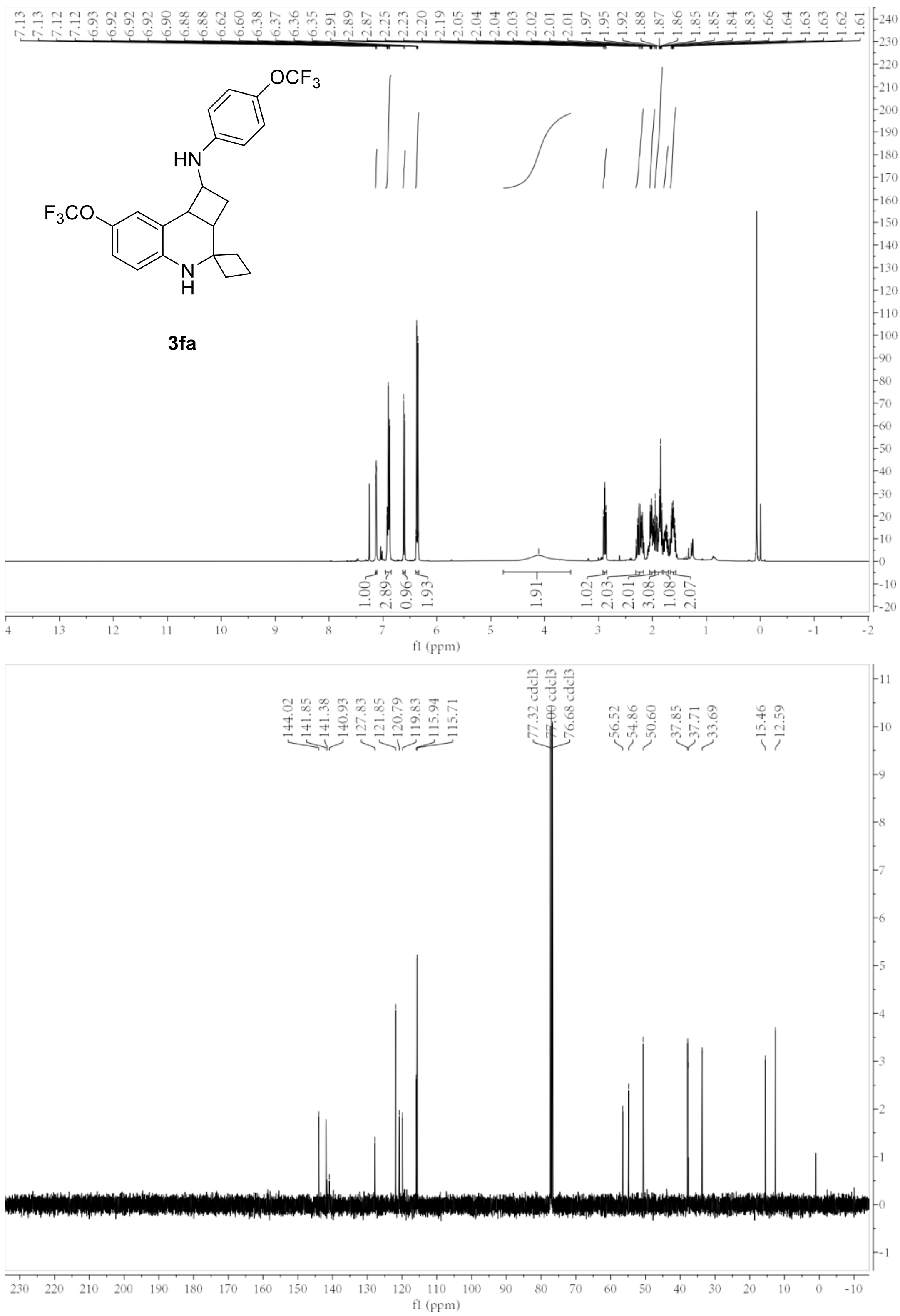

Figure S6. ^{1}H (400 MHz, $CDCl_3$) and ^{13}C (101 MHz, $CDCl_3$) NMR spectra for compound **3fa**

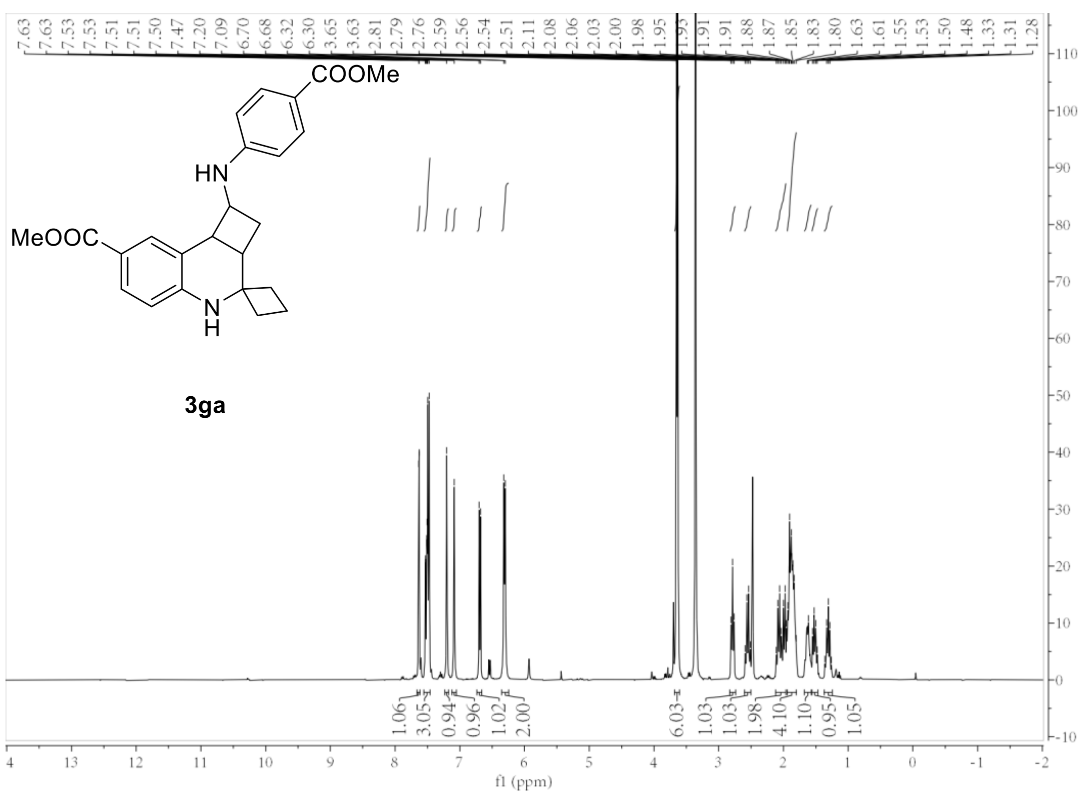

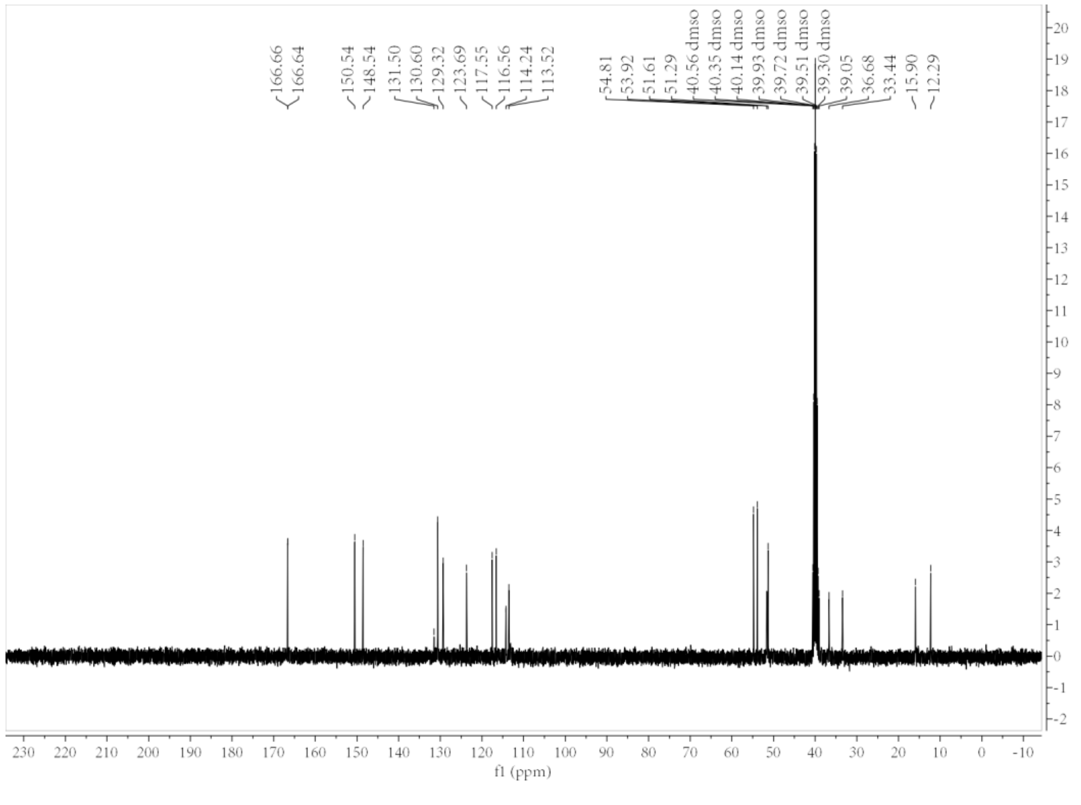

Figure S7. ^{1}H (400 MHz, DMSO) and ^{13}C (101 MHz, DMSO) NMR spectra for compound **3ga**

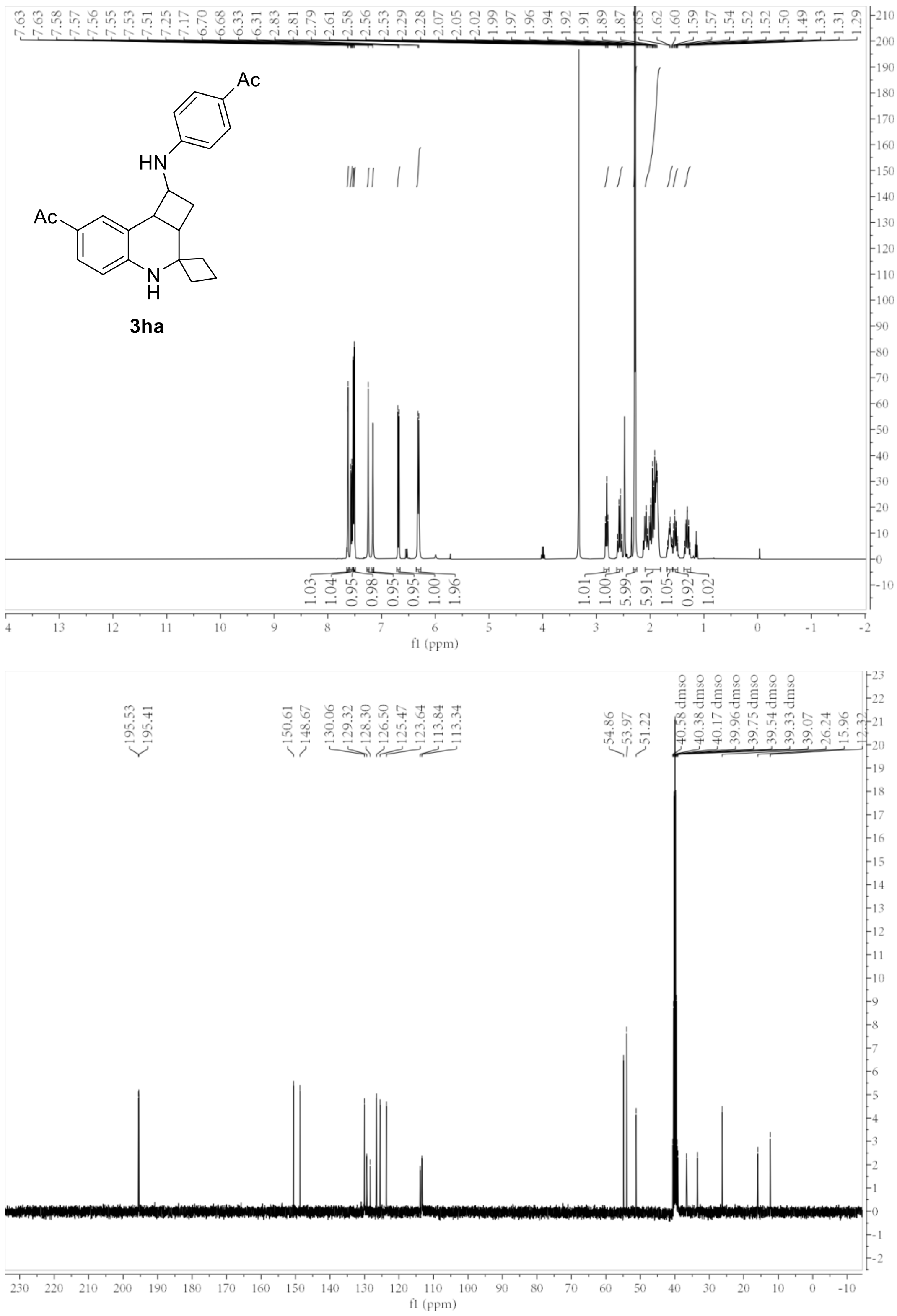

Figure S8. ^{1}H (400 MHz, DMSO) and ^{13}C (101 MHz, DMSO) NMR spectra for compound **3ha**

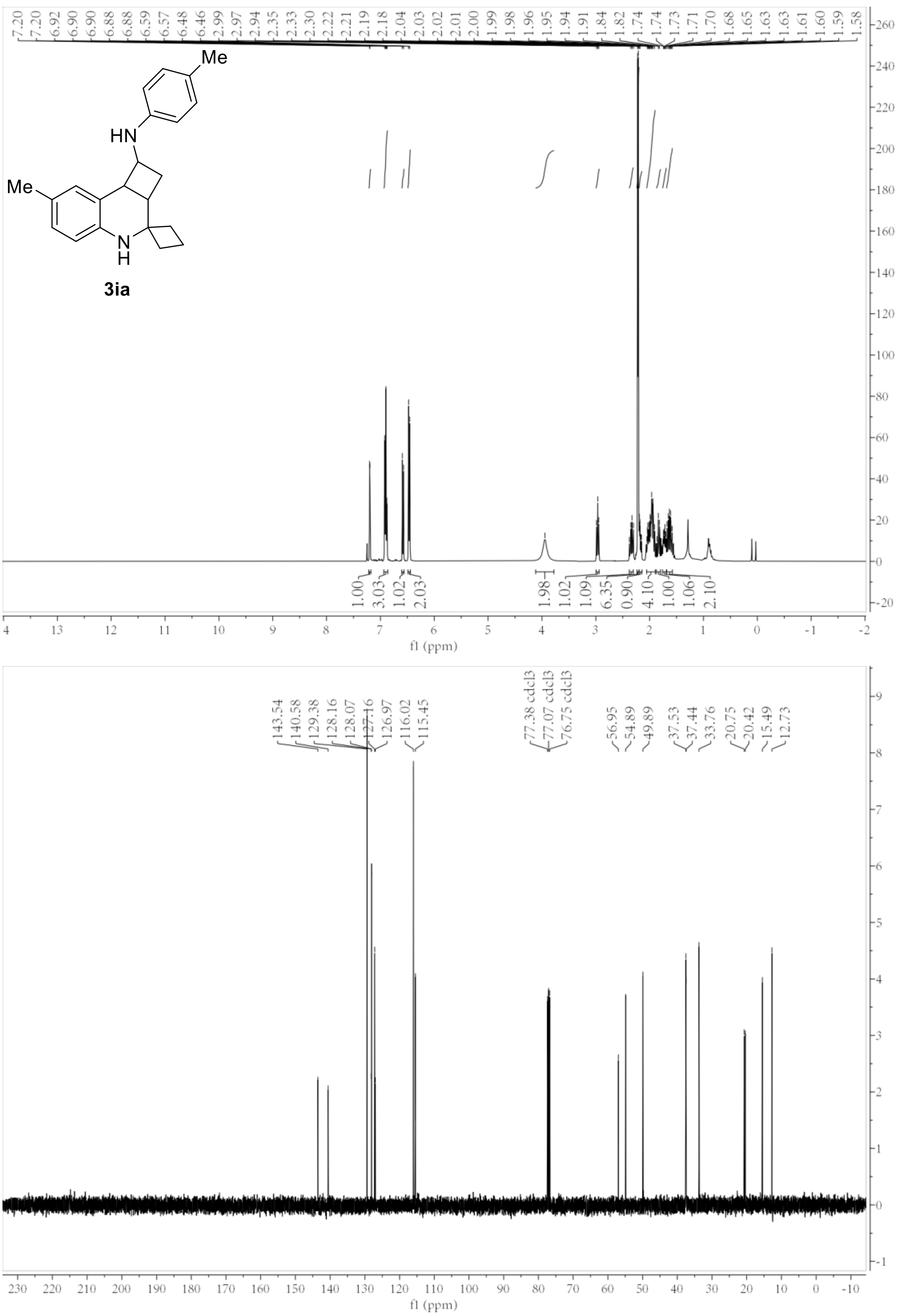

Figure S9. ^{1}H (400 MHz, $CDCl_3$) and ^{13}C (101 MHz, $CDCl_3$) NMR spectra for compound **3ia**

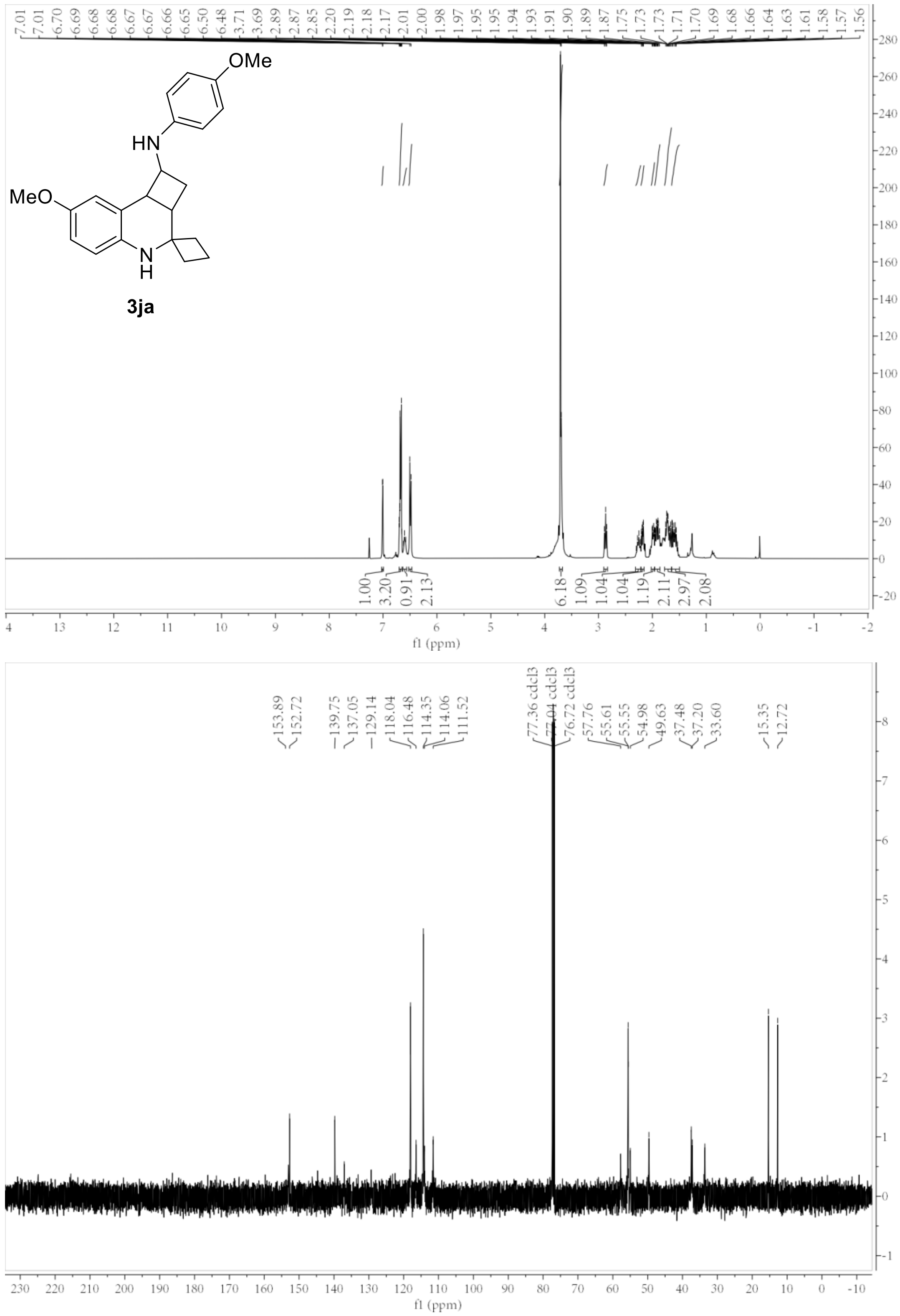

Figure S10. ^{1}H (400 MHz, $CDCl_3$) and ^{13}C (101 MHz, $CDCl_3$) NMR spectra for compound **3*J*a**

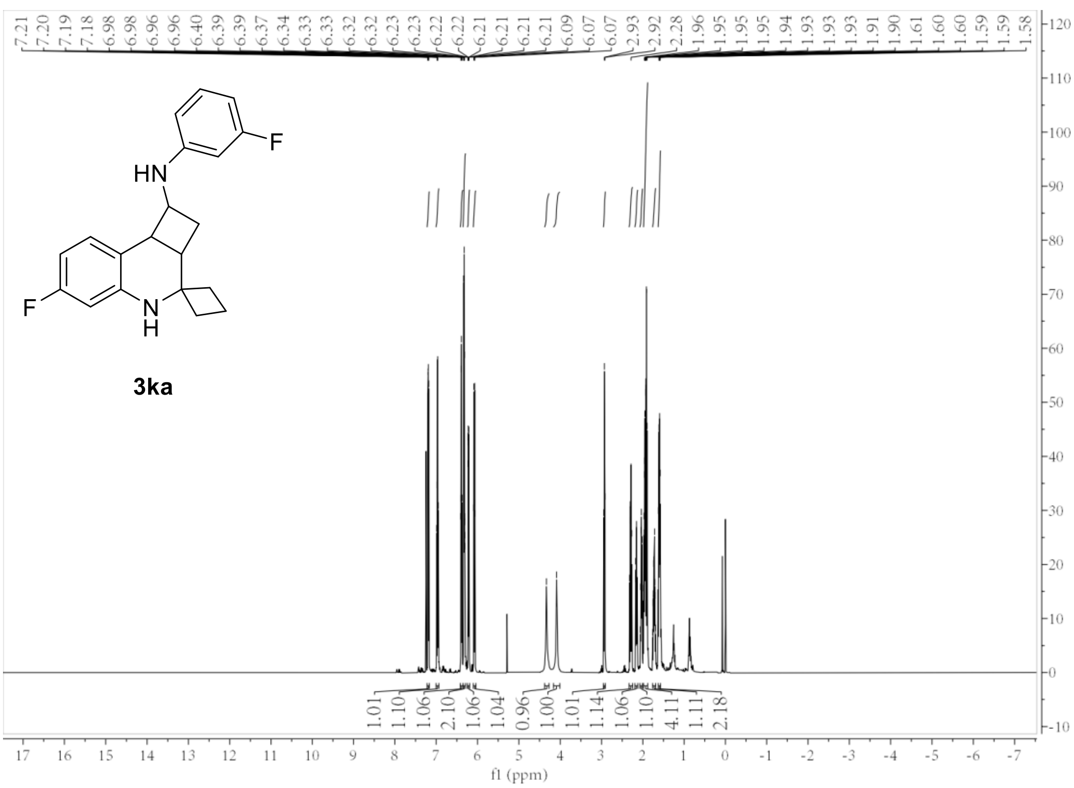

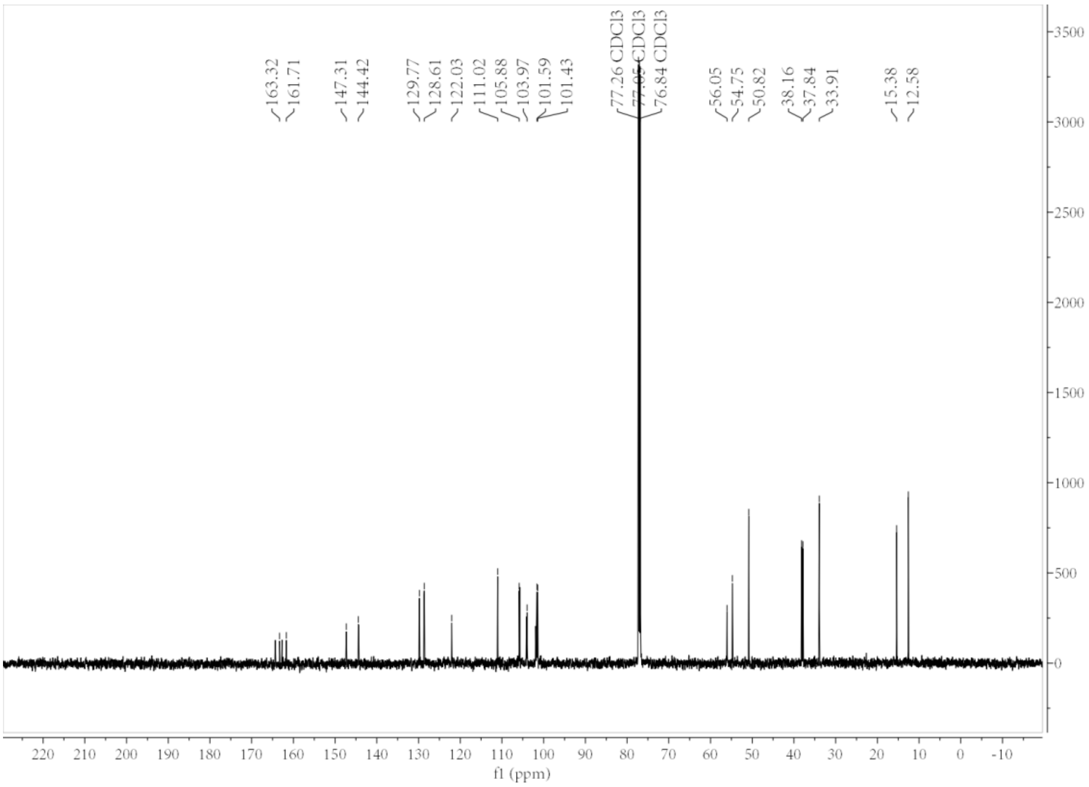

Figure S11. ^{1}H (400 MHz, $CDCl_3$) and ^{13}C (101 MHz, $CDCl_3$) NMR spectra for compound **3ka**

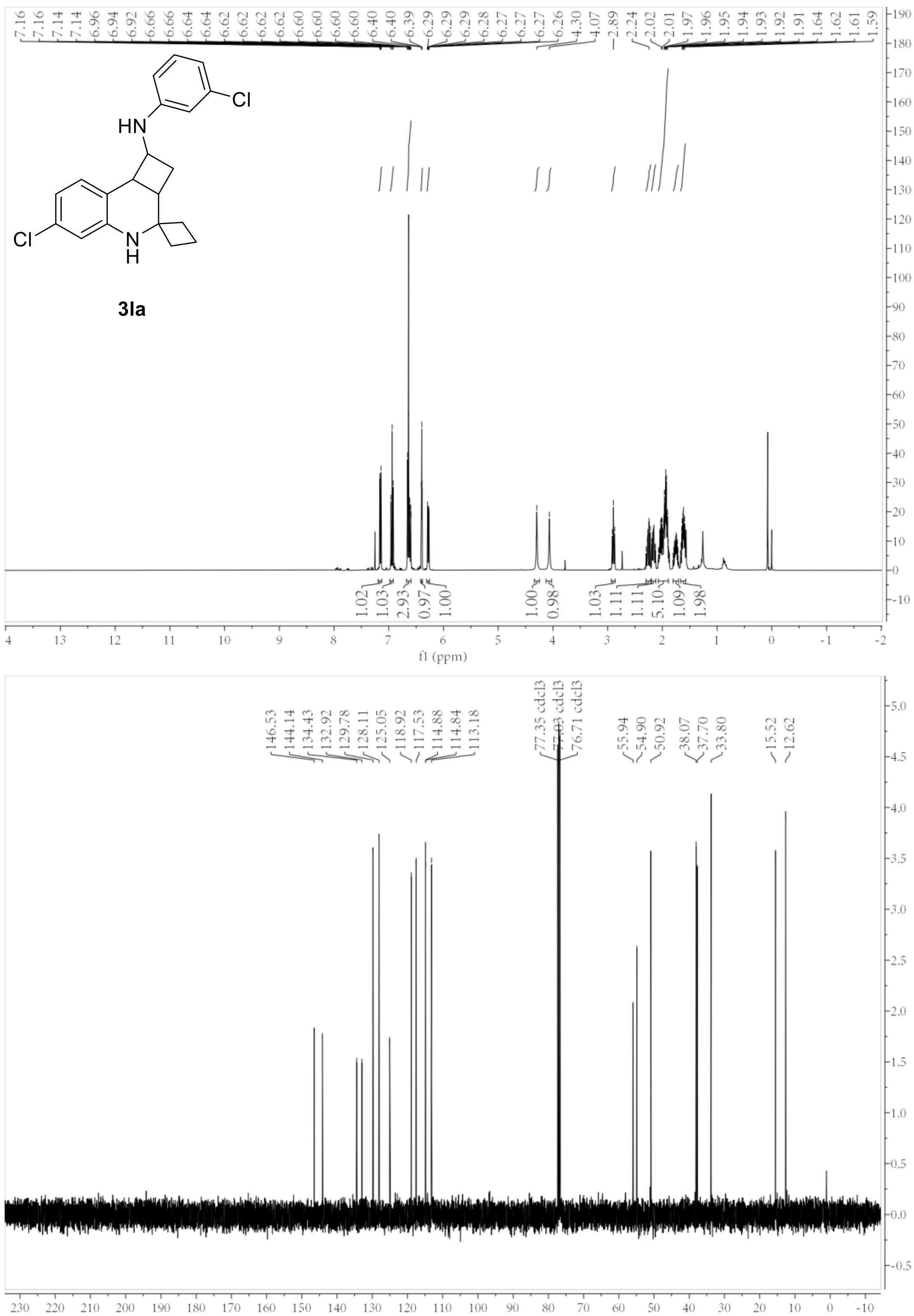

Figure S12. ^{1}H (400 MHz, $CDCl_3$) and ^{13}C (101 MHz, $CDCl_3$) NMR spectra for compound **3la**

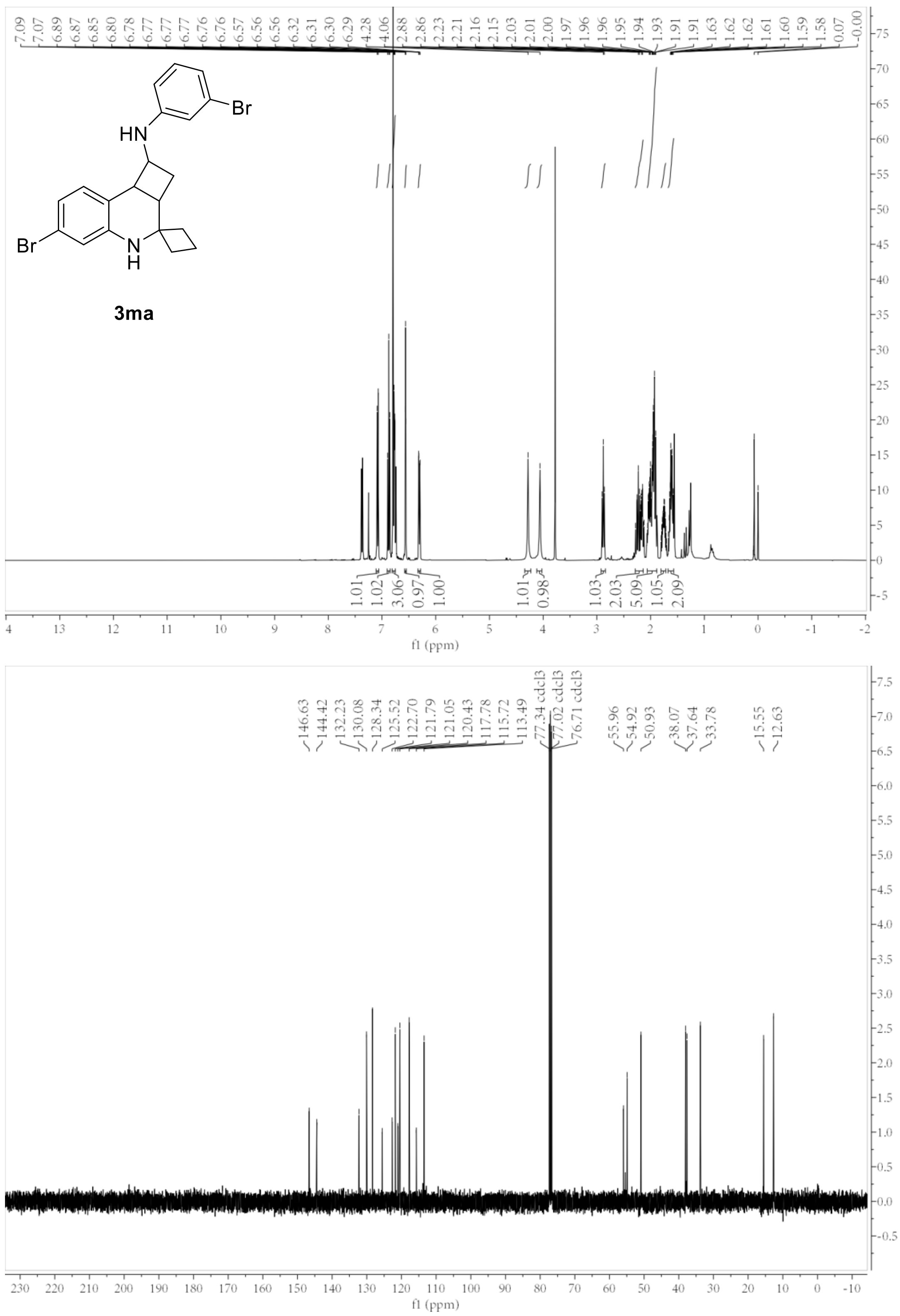

Figure S13. ^{1}H (400 MHz, $CDCl_3$) and ^{13}C (101 MHz, $CDCl_3$) NMR spectra for compound **3ma**

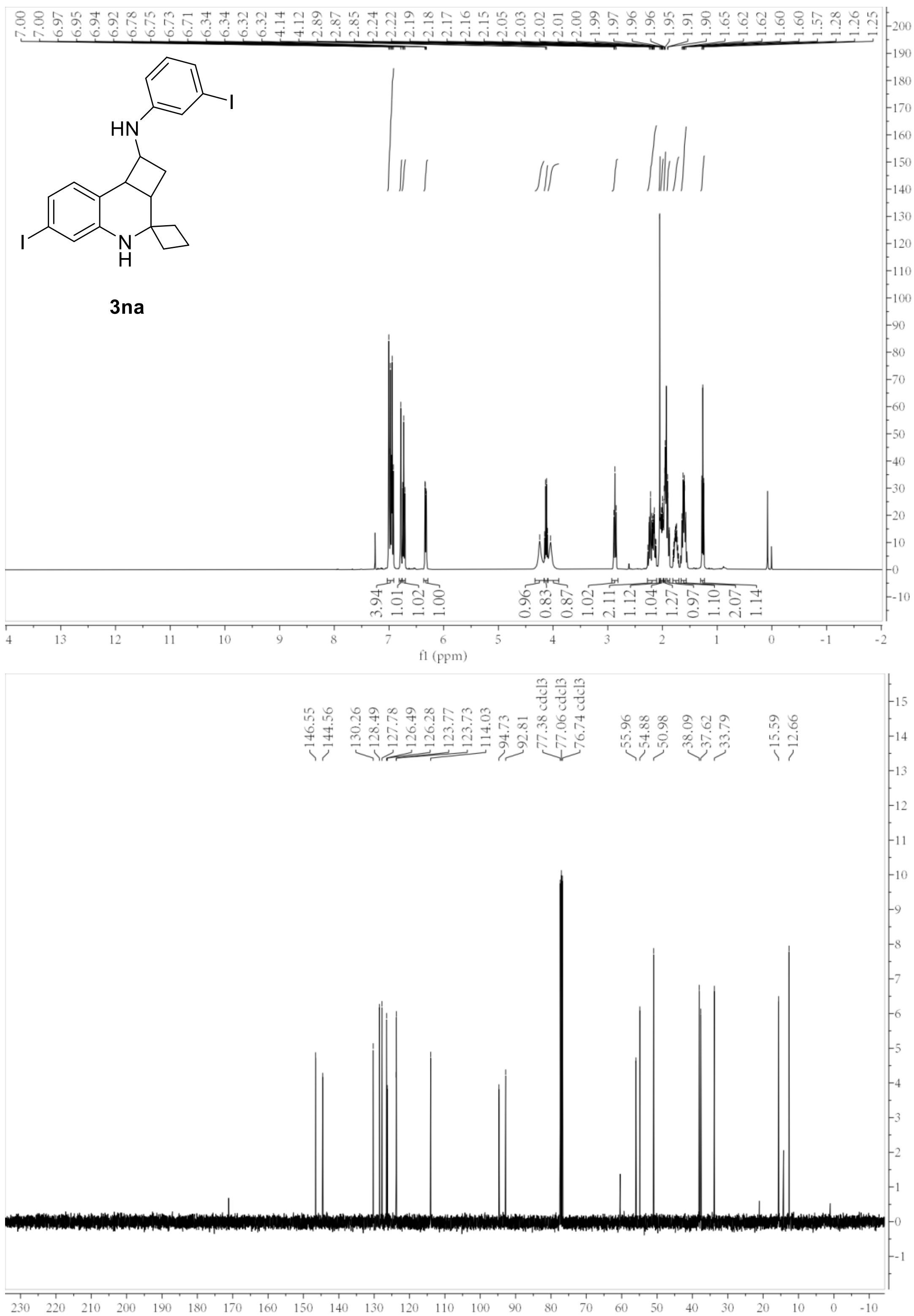

Figure S14. ^{1}H (400 MHz, $CDCl_3$) and ^{13}C (101 MHz, $CDCl_3$) NMR spectra for compound **3na**

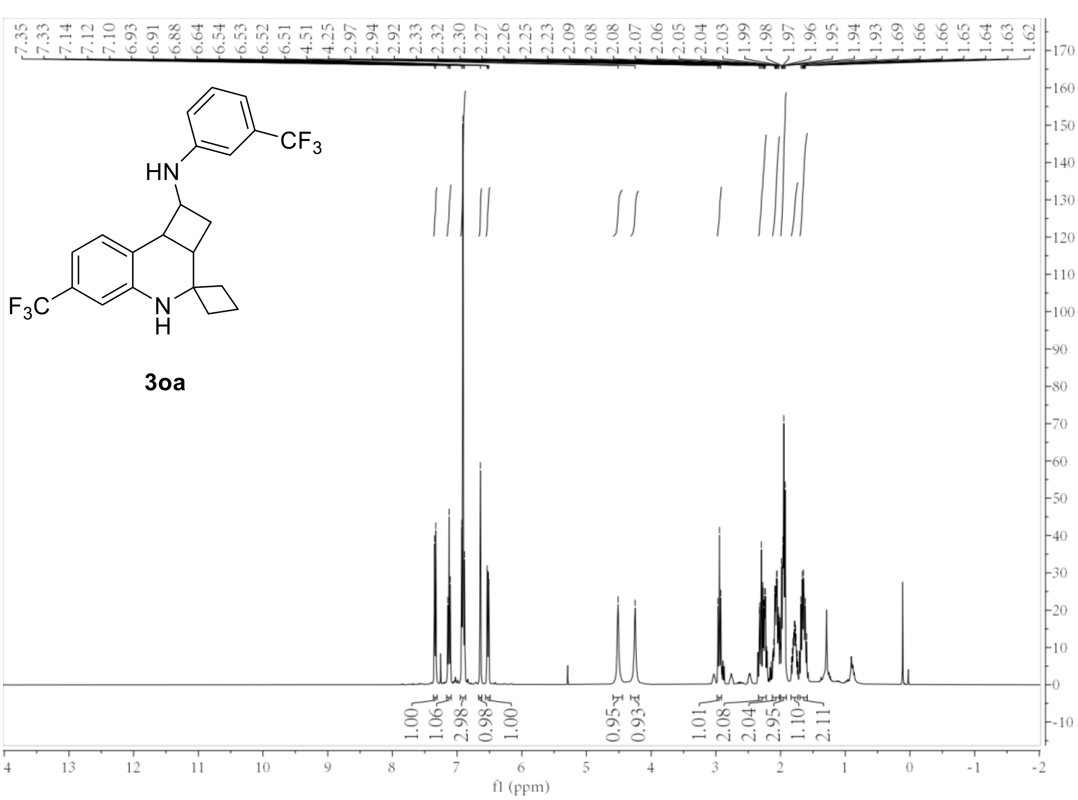

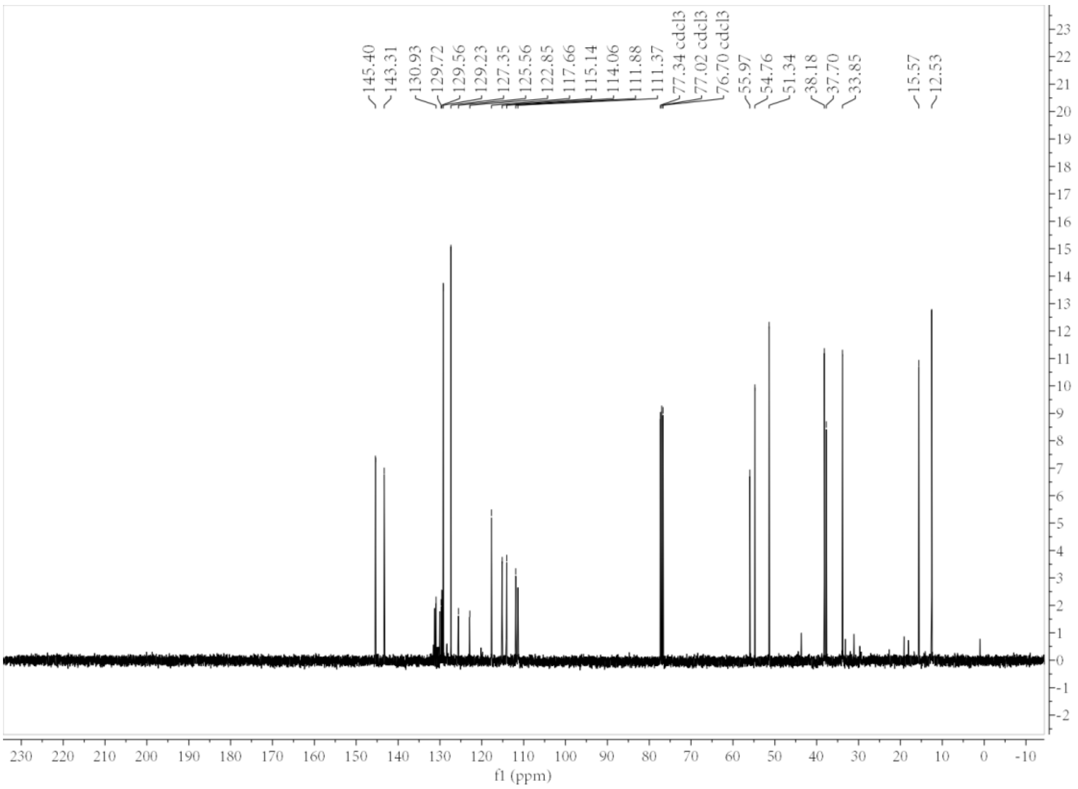

Figure S15. ^{1}H (400 MHz, $CDCl_3$) and ^{13}C (101 MHz, $CDCl_3$) NMR spectra for compound **3oa**

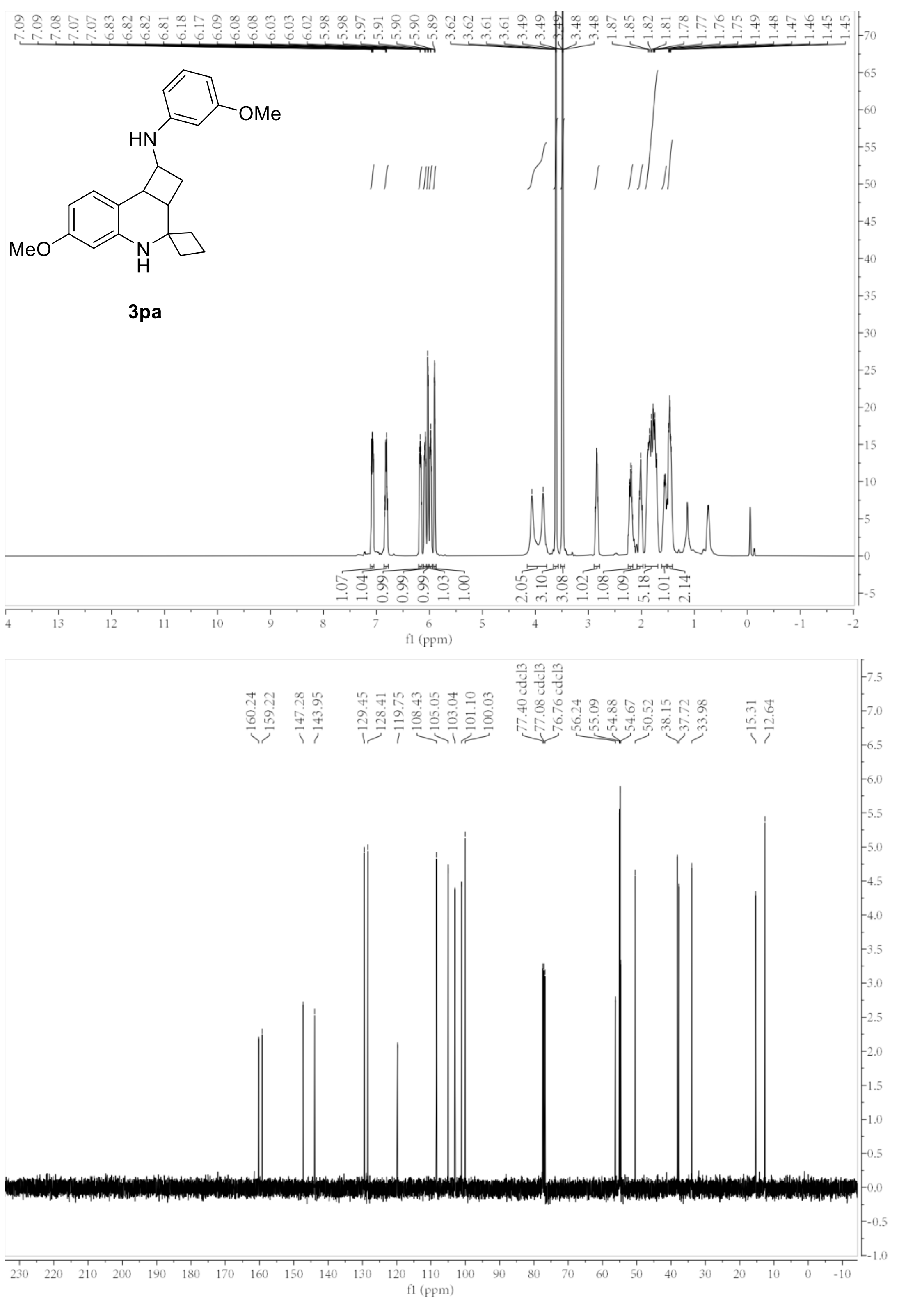

Figure S16. ^{1}H (400 MHz, $CDCl_3$) and ^{13}C (101 MHz, $CDCl_3$) NMR spectra for compound **3pa**

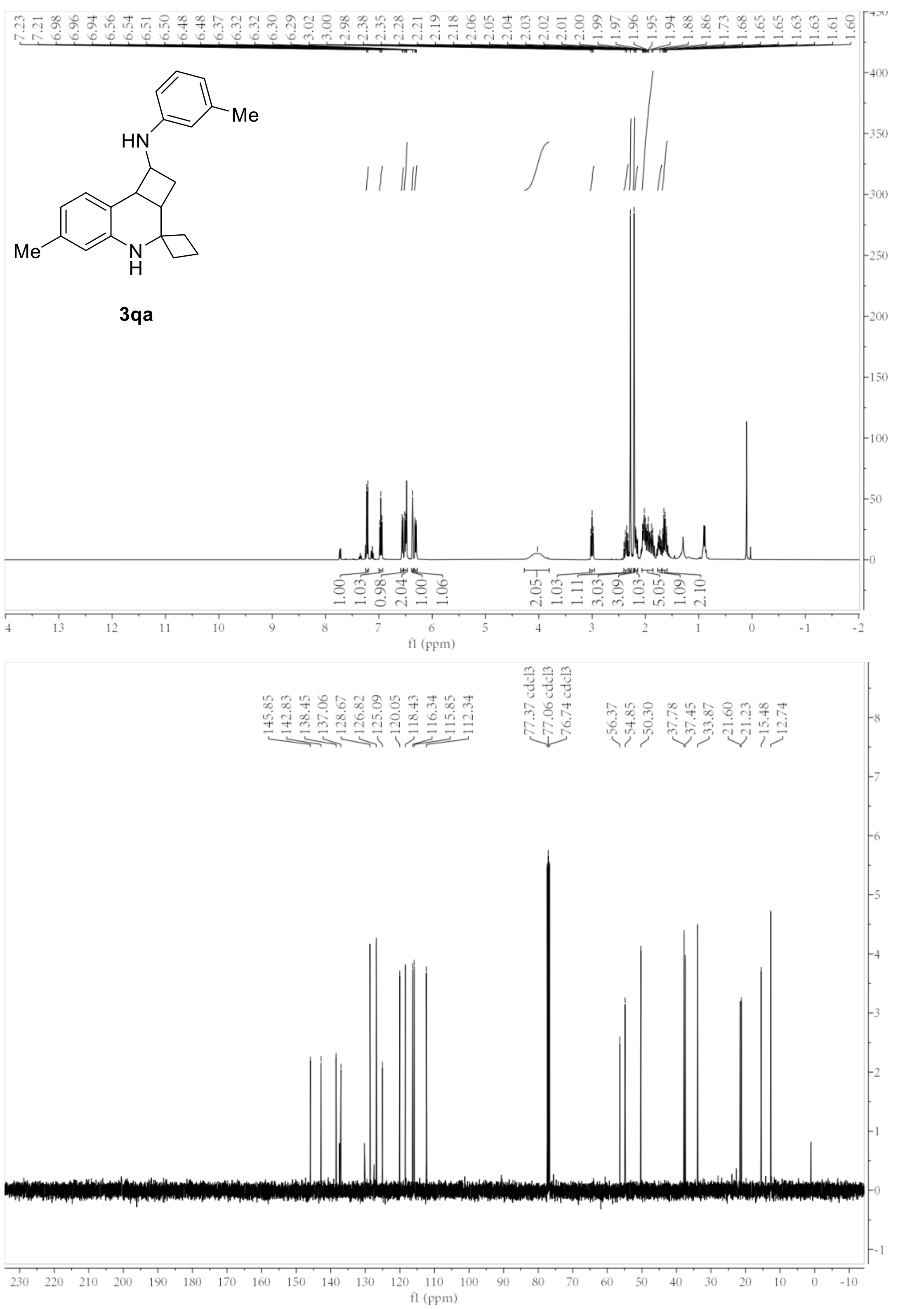

Figure S17. ^{1}H (400 MHz, $CDCl_3$) and ^{13}C (101 MHz, $CDCl_3$) NMR spectra for compound **3qa**

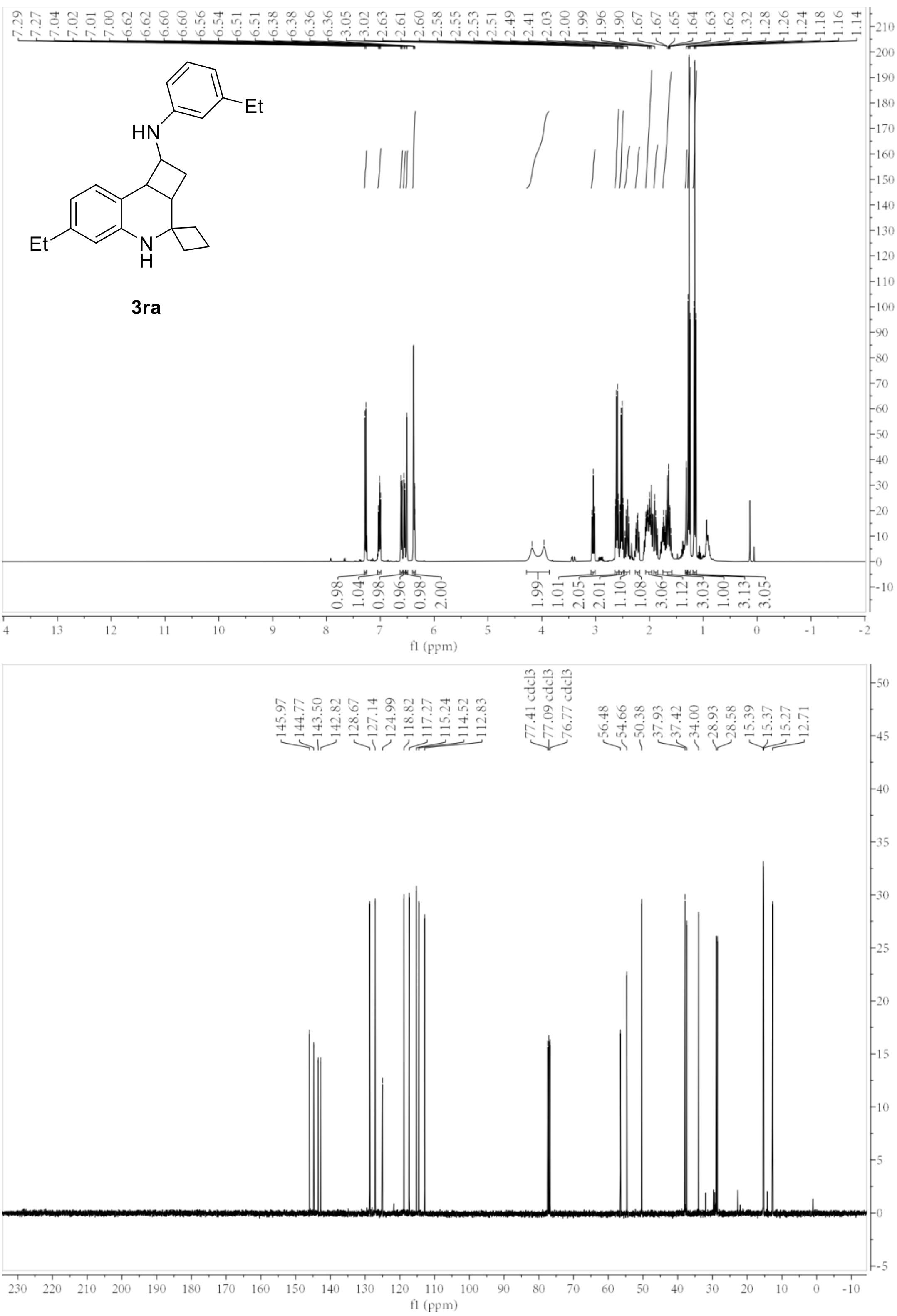

Figure S18. ^{1}H (400 MHz, $CDCl_3$) and ^{13}C (101 MHz, $CDCl_3$) NMR spectra for compound **3ra**

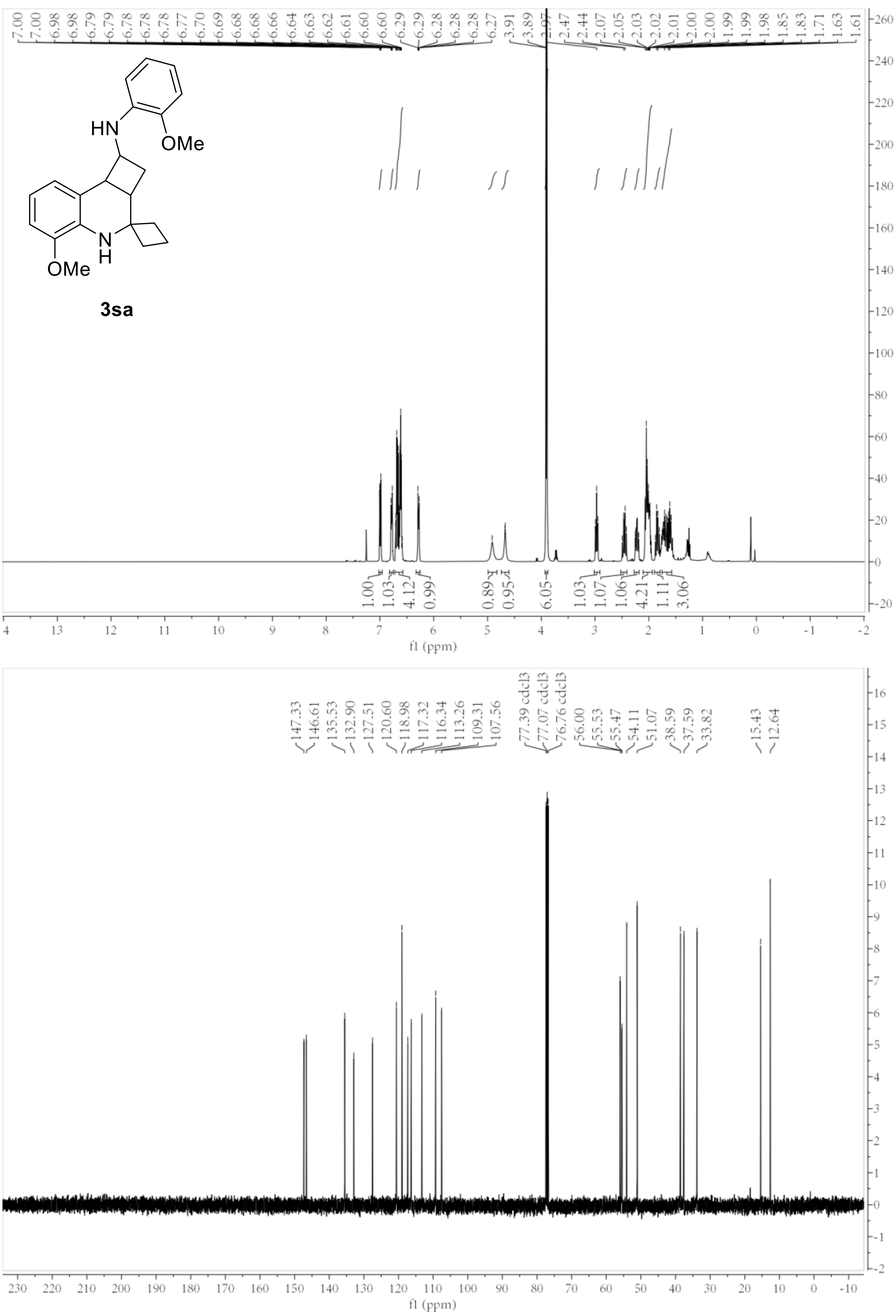

Figure S19. ^{1}H (400 MHz, $CDCl_3$) and ^{13}C (101 MHz, $CDCl_3$) NMR spectra for compound **3sa**

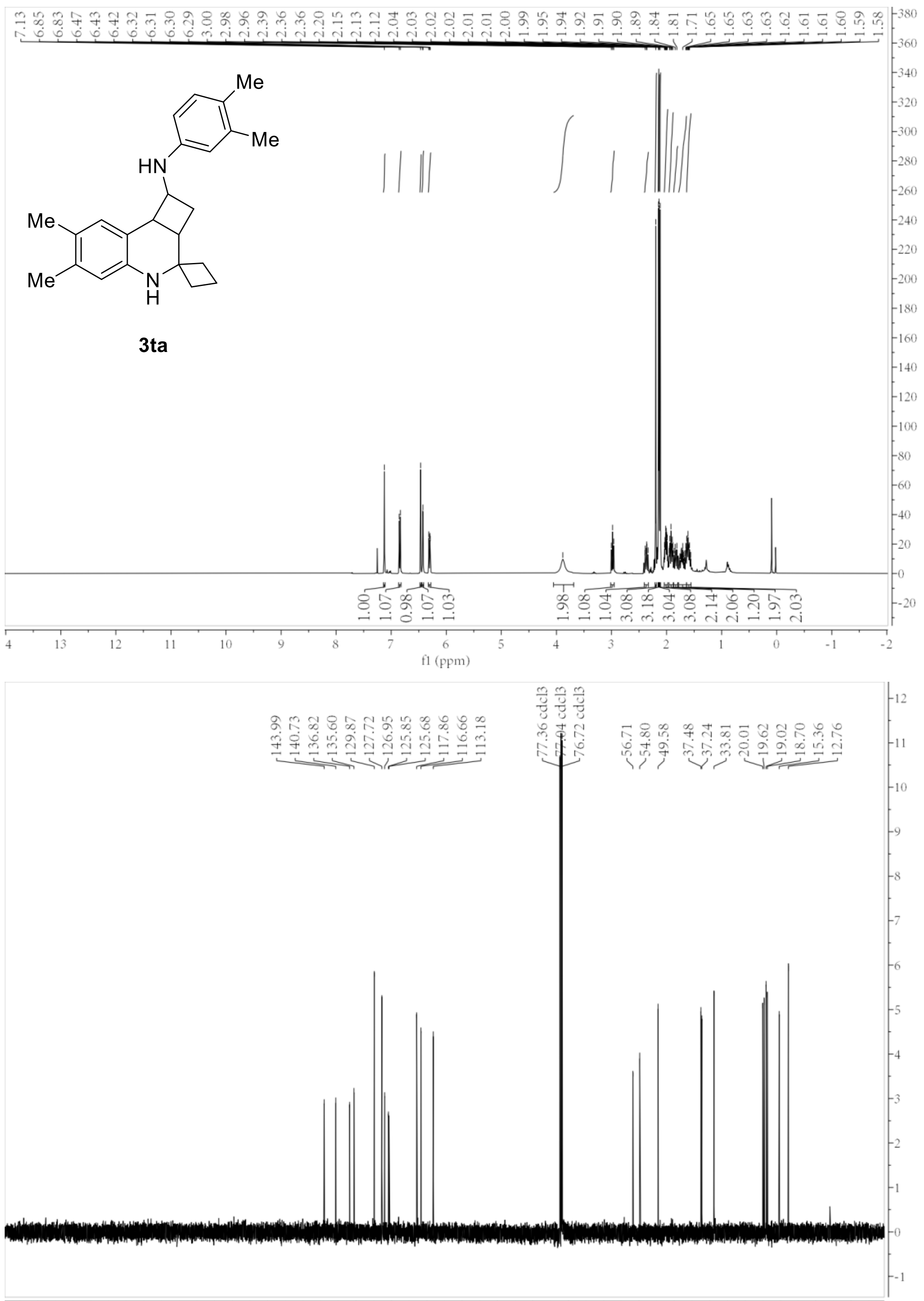

Figure S20. ^{1}H (400 MHz, $CDCl_3$) and ^{13}C (101 MHz, $CDCl_3$) NMR spectra for compound **3ta**

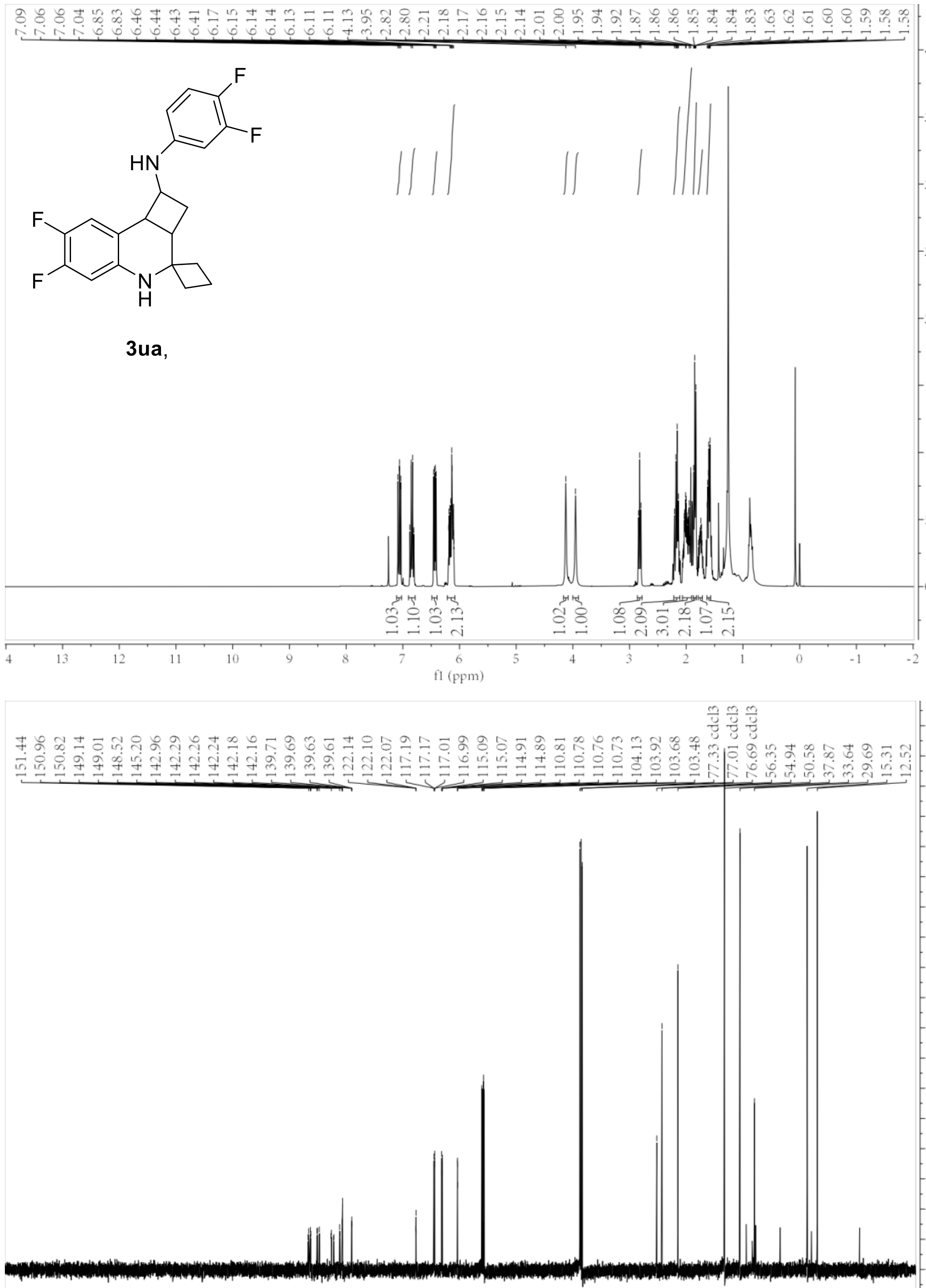

Figure S21. ^{1}H (400 MHz, $CDCl_3$) and ^{13}C (101 MHz, $CDCl_3$) NMR spectra for compound **3ua**

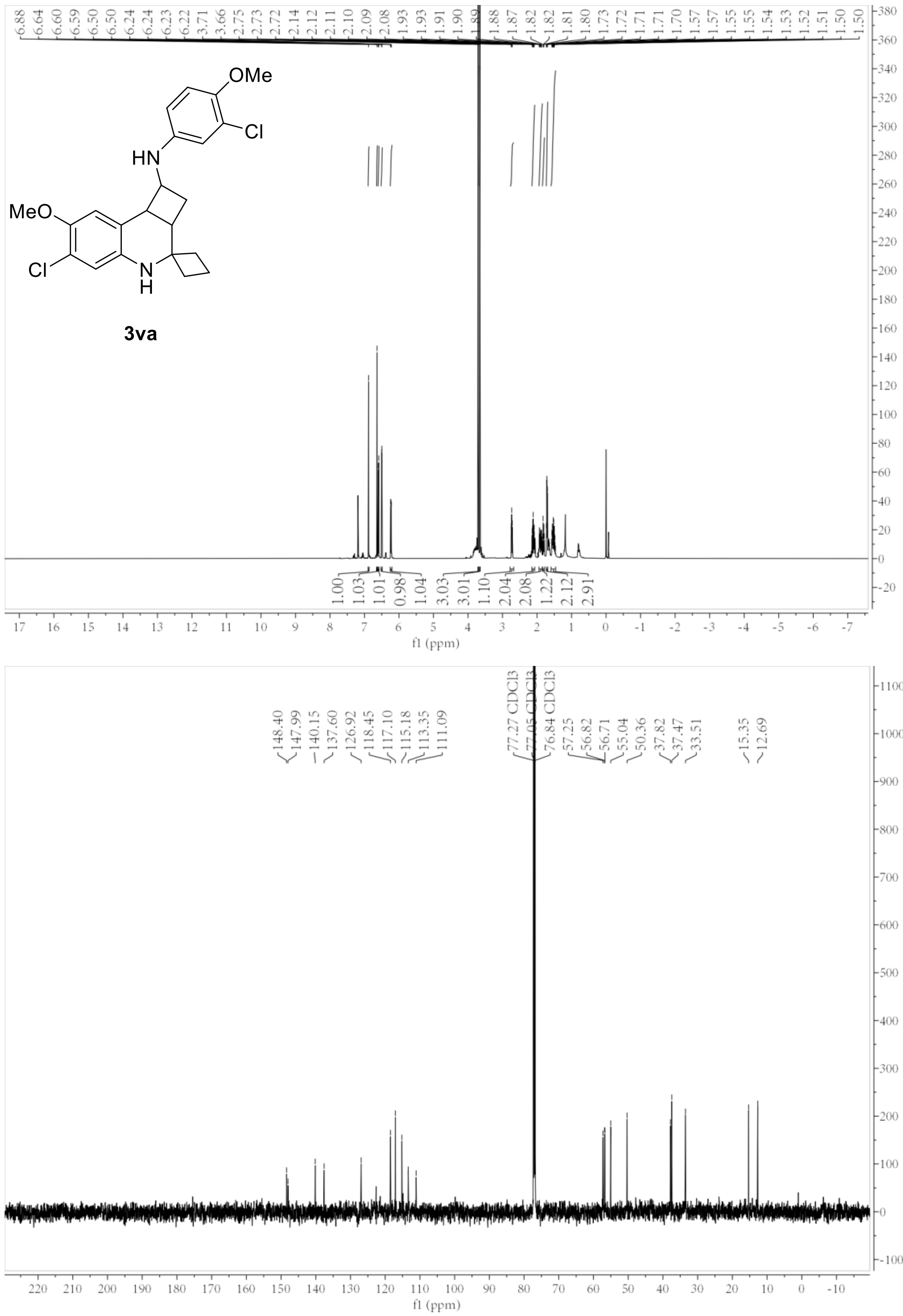

Figure S22. ^{1}H (400 MHz, $CDCl_3$) and ^{13}C (101 MHz, $CDCl_3$) NMR spectra for compound **3va**

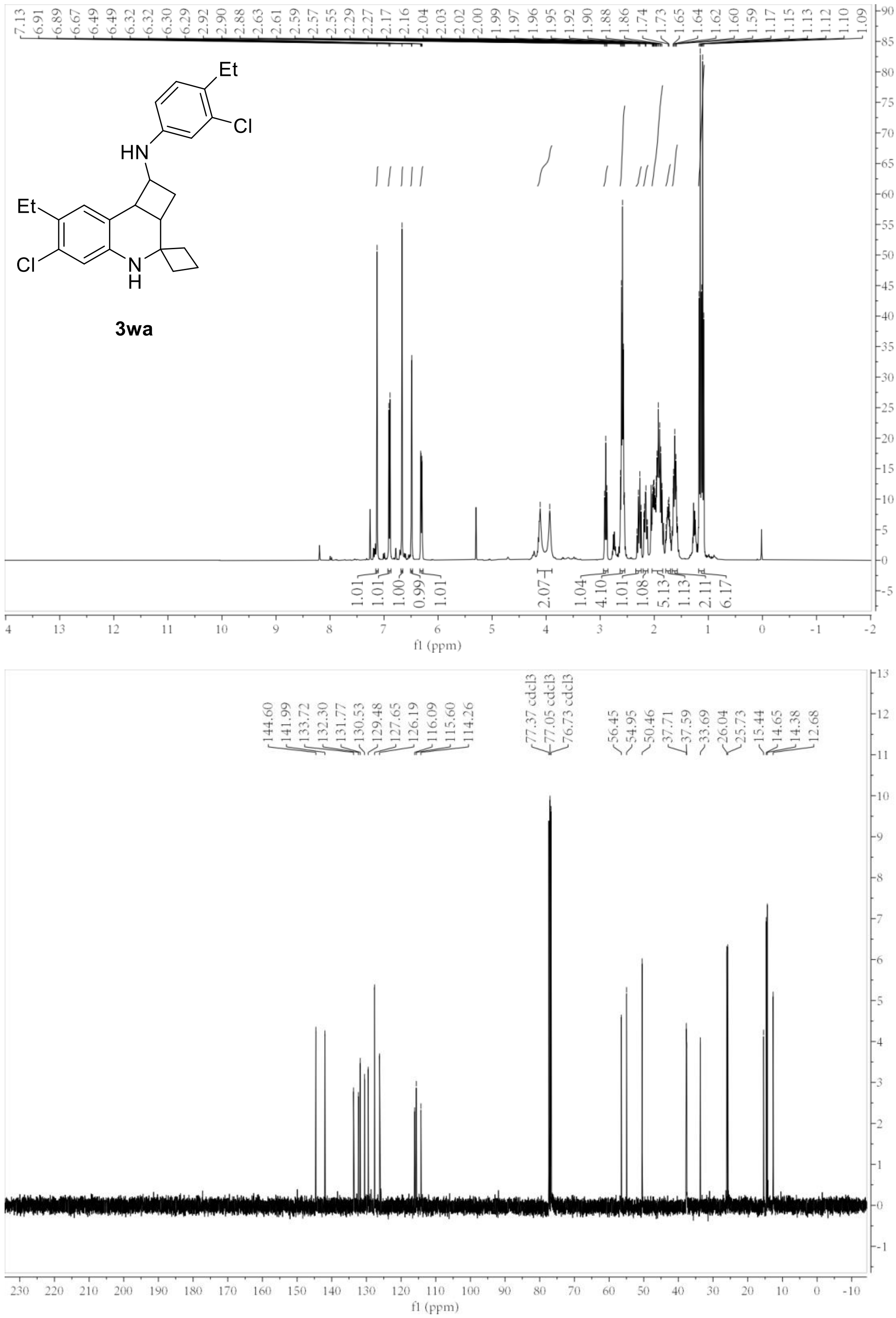

Figure S23. ^{1}H (400 MHz, $CDCl_3$) and ^{13}C (101 MHz, $CDCl_3$) NMR spectra for compound **3wa**

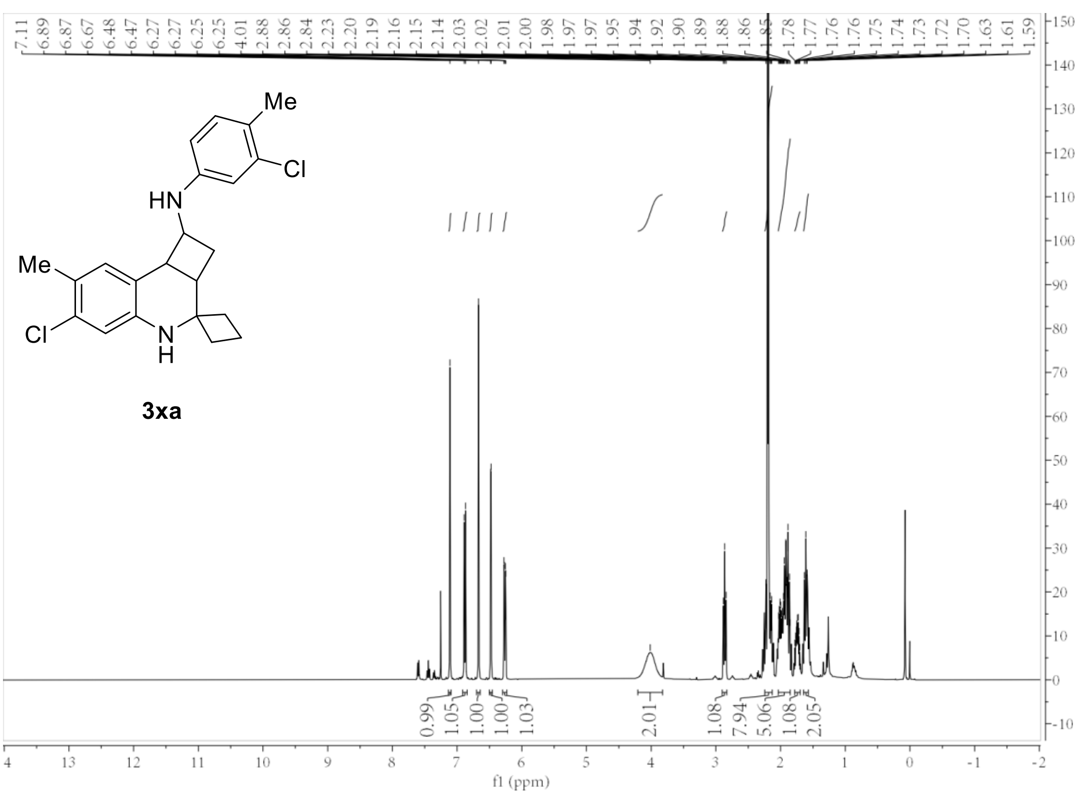

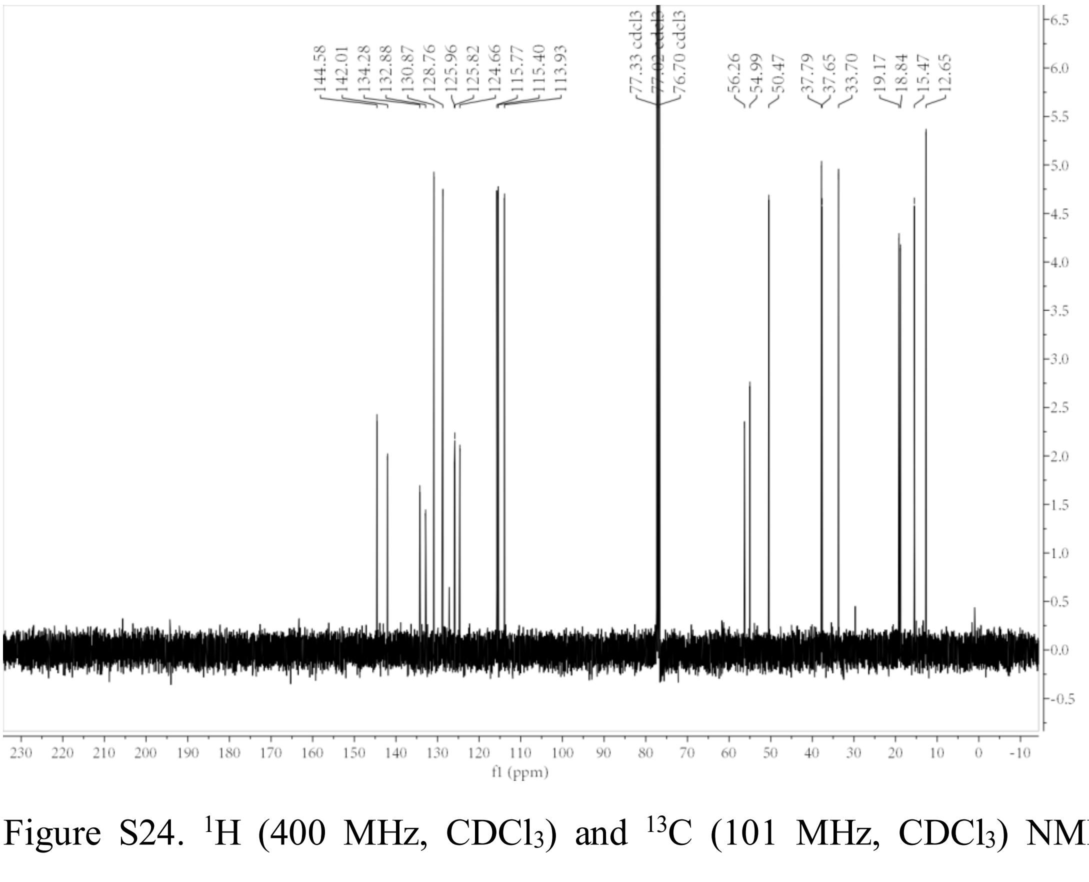

Figure S24. ^{1}H (400 MHz, $CDCl_3$) and ^{13}C (101 MHz, $CDCl_3$) NMR spectra for compound **3xa**

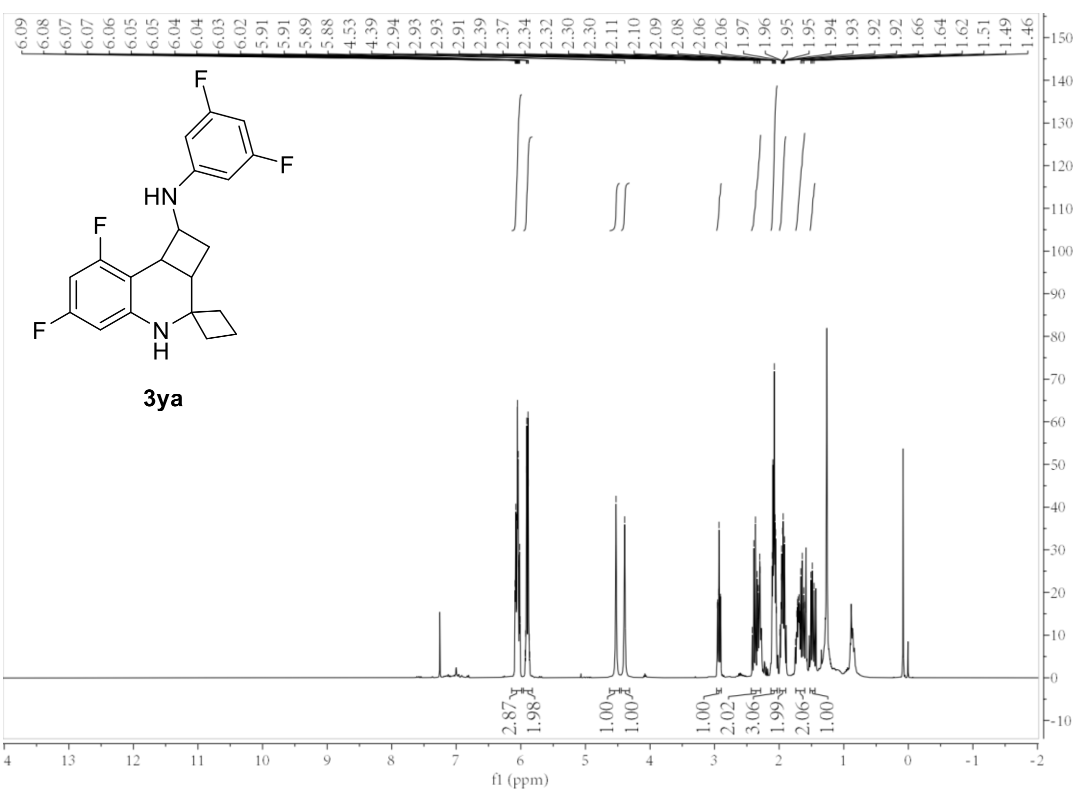

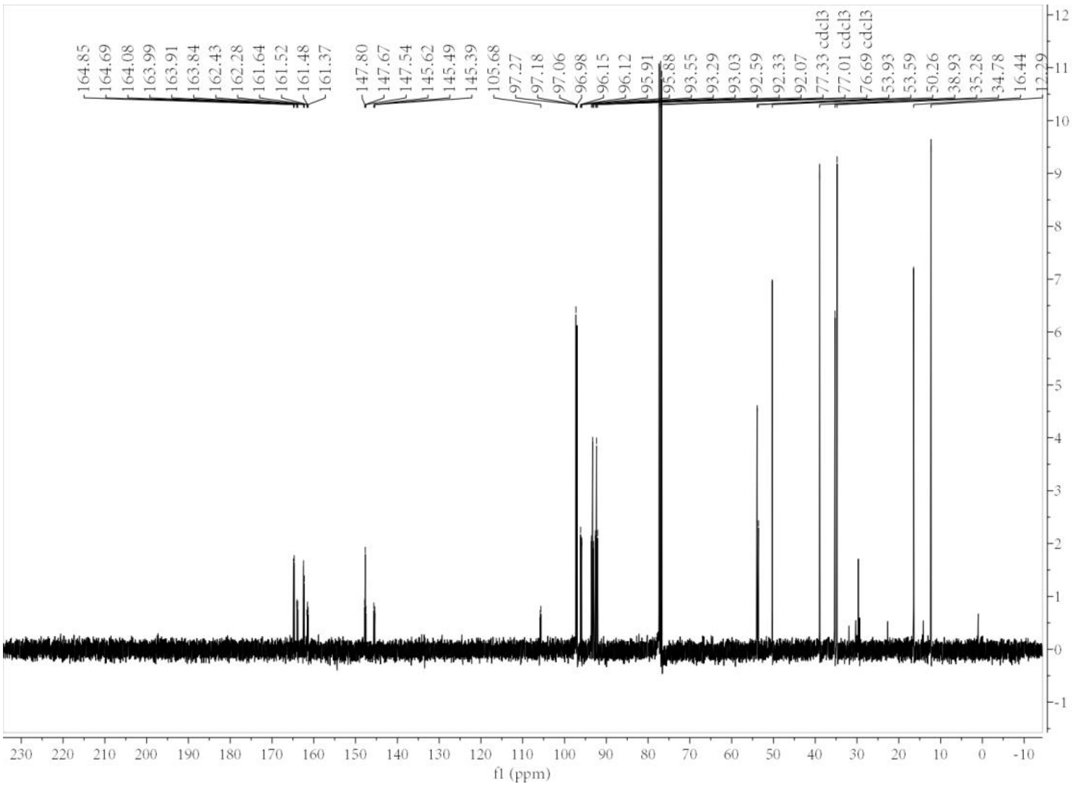

Figure S25. ^{1}H (400 MHz, $CDCl_3$) and ^{13}C (101 MHz, $CDCl_3$) NMR spectra for compound **3ya**

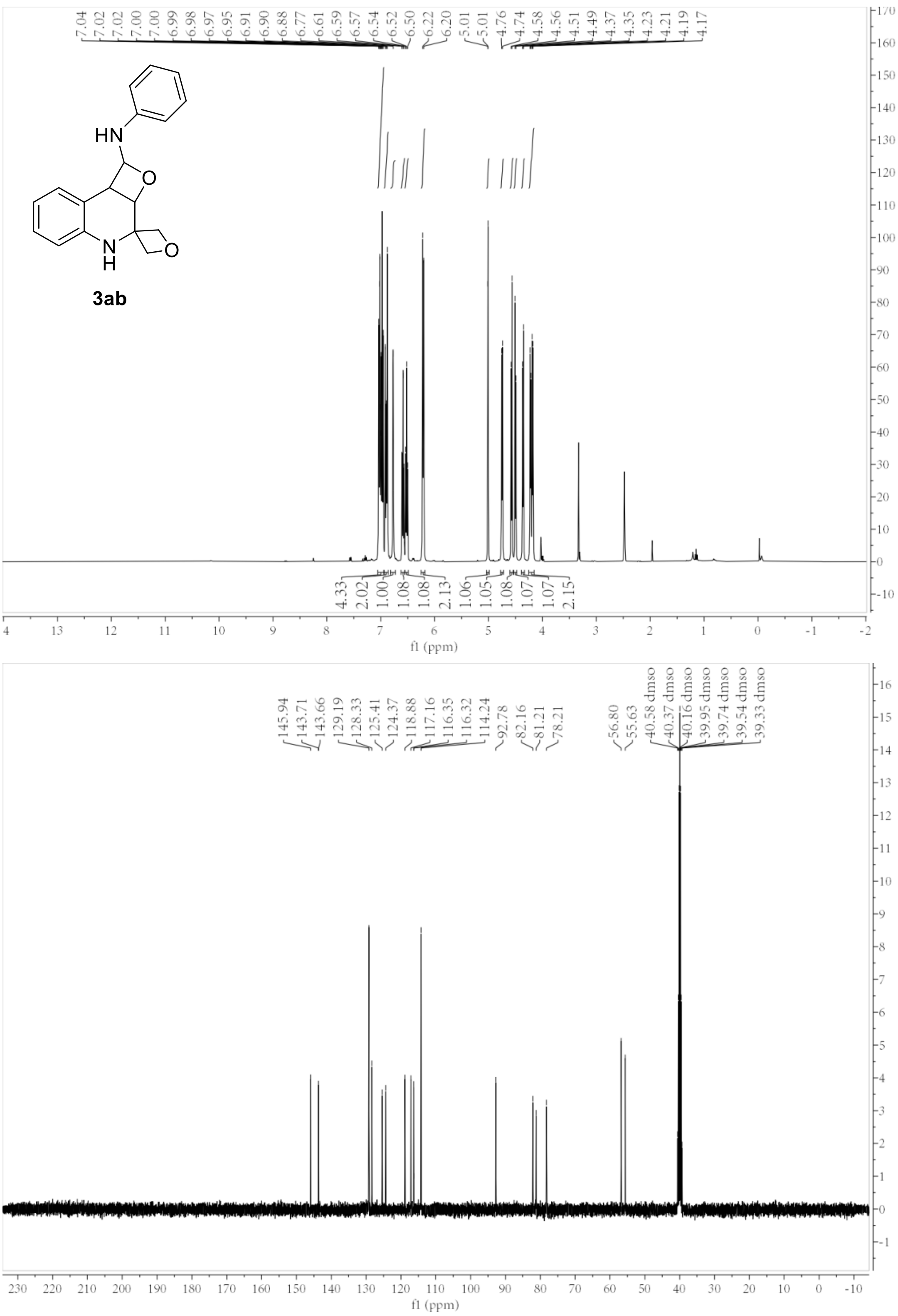

Figure S26. ^{1}H (400 MHz, DMSO) and ^{13}C (101 MHz, DMSO) NMR spectra for compound **3ab**

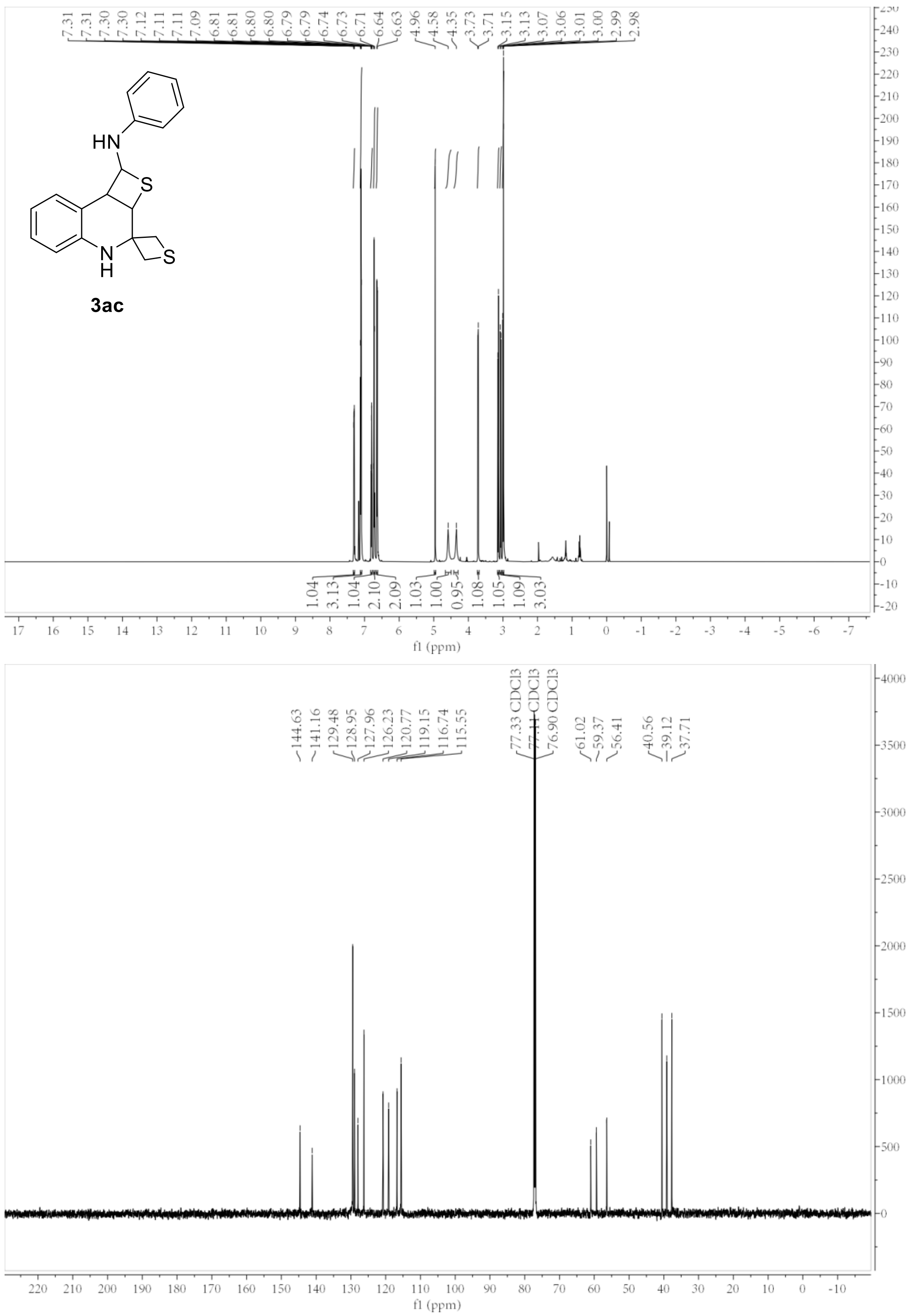

Figure S27. ^{1}H (400 MHz, $CDCl_3$) and ^{13}C (101 MHz, $CDCl_3$) NMR spectra for compound **3ac**

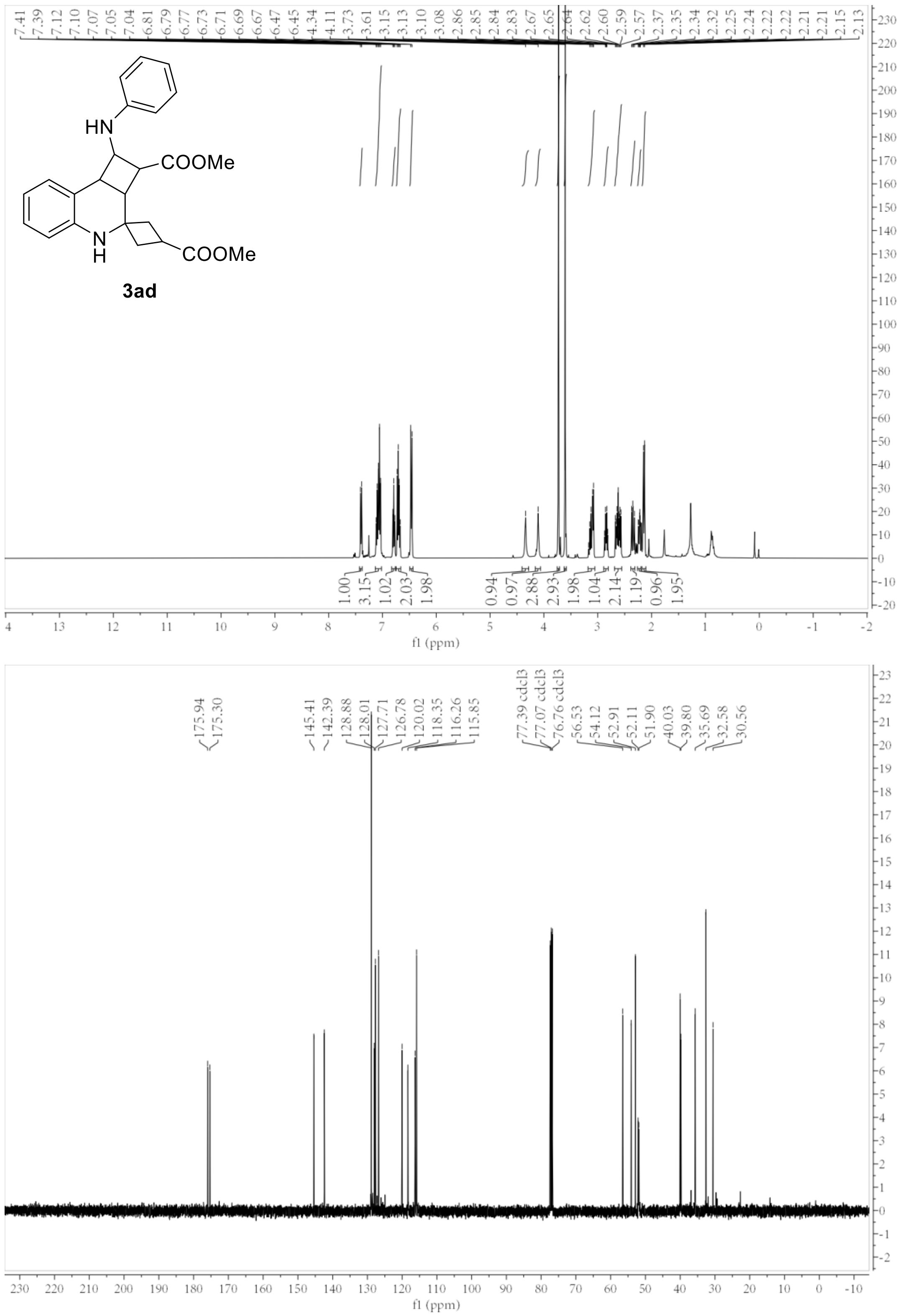

Figure S28. ^{1}H (400 MHz, $CDCl_3$) and ^{13}C (101 MHz, $CDCl_3$) NMR spectra for compound **3ad**

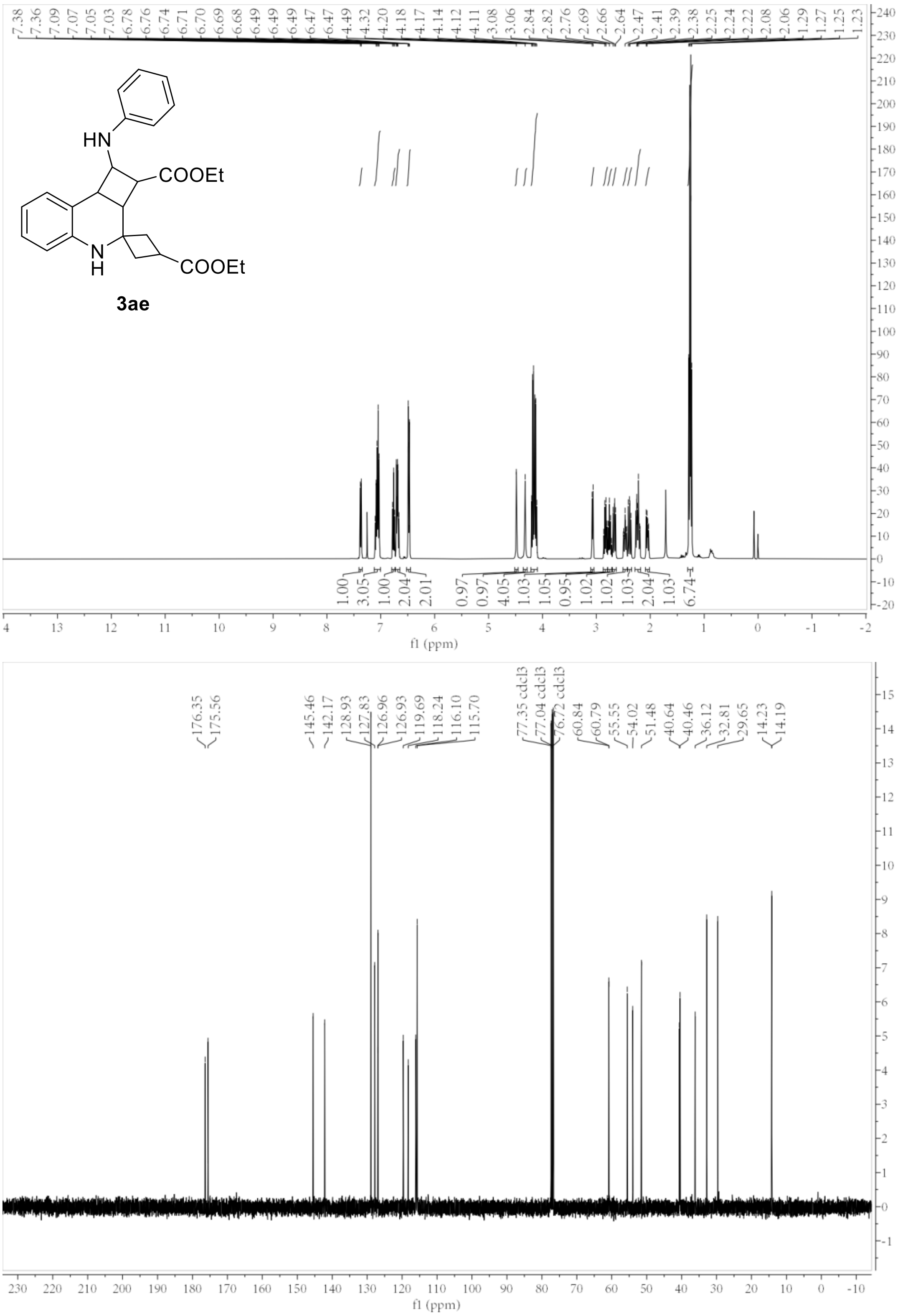

Figure S28. ^{1}H (400 MHz, $CDCl_3$) and ^{13}C (101 MHz, $CDCl_3$) NMR spectra for compound **3ad**
